# Single molecule-level detection and long read-based phasing of epigenetic variations in bacterial methylomes

**DOI:** 10.1038/ncomms8438

**Published:** 2015-06-15

**Authors:** John Beaulaurier, Xue-Song Zhang, Shijia Zhu, Robert Sebra, Chaggai Rosenbluh, Gintaras Deikus, Nan Shen, Diana Munera, Matthew K. Waldor, Andrew Chess, Martin J. Blaser, Eric E. Schadt, Gang Fang

**Affiliations:** 1 Department of Genetics and Genomic Sciences and Icahn Institute for Genomics and Multiscale Biology, Icahn School of Medicine at Mount Sinai, New York 10029, USA; 2 Department of Medicine, New York University School of Medicine, New York 10016, USA; 3 Division of Infectious Diseases, Brigham and Women's Hospital, Harvard Medical School, and the Howard Hughes Medical Institute, Boston, Massachusetts 02115, USA

## Abstract

Beyond its role in host defense, bacterial DNA methylation also plays important roles in the regulation of gene expression, virulence and antibiotic resistance. Bacterial cells in a clonal population can generate epigenetic heterogeneity to increase population-level phenotypic plasticity. Single molecule, real-time (SMRT) sequencing enables the detection of N6-methyladenine and N4-methylcytosine, two major types of DNA modifications comprising the bacterial methylome. However, existing SMRT sequencing-based methods for studying bacterial methylomes rely on a population-level consensus that lacks the single-cell resolution required to observe epigenetic heterogeneity. Here, we present SMALR (single-molecule modification analysis of long reads), a novel framework for single molecule-level detection and phasing of DNA methylation. Using seven bacterial strains, we show that SMALR yields significantly improved resolution and reveals distinct types of epigenetic heterogeneity. SMALR is a powerful new tool that enables *de novo* detection of epigenetic heterogeneity and empowers investigation of its functions in bacterial populations.

## Abstract (web summary)

Bacterial DNA methylation is involved in many processes, from host defense to antibiotic resistance, however current methods for examining methylated genomes lack single-cell resolution. Here Beaulaurier *et al.* present Single Molecule Modification Analysis of Long Reads, a new tool for *de novo* detection of epigenetic heterogeneity.

## Graphical abstract

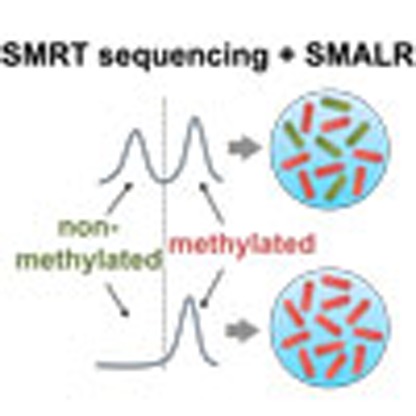

In the bacterial kingdom, DNA methylation is catalysed by three families of DNA methyltransferases (MTases) that typically add methyl groups to DNA bases in a sequence-specific manner^1^,^2^,^3^. One family of MTases attaches methyl groups to adenine residues, creating N6-methyladenine (6mA), whereas the other two families introduce methyl groups to cytosine residues to create either N4-methylcytosine (4mC) or 5-methylcytosine (5mC). Many bacterial DNA MTases act in concert with and are encoded in close proximity to cognate restriction endonucleases (REs); the MTase protects DNA from digestion by the RE with which it forms a restriction-modification (R-M) system^2^,^4^,^5^. R-M systems are generally believed to have an ‘immune' function, protecting cells from invading foreign DNA. They are also studied as selfish elements that protect themselves against removal through the post-segregational killing of new progeny by pre-existing and stable RE molecules^4^,^6^,^7^. In addition, so-called ‘orphan' MTases, which occur in the genome without an associated RE, have been found to play important regulatory roles in global gene expression and other biological processes^2^,^3^,^8^,^9^,^10^. Furthermore, the ability of such MTases to target their recognition motifs for methylation often depends on competitive binding at the target site between several DNA-binding proteins^2^,^3^,^11^,^12^,^13^,^14^,^15^. These epigenetic regulators of gene expression, including both MTases and competing DNA-binding proteins, are a source of phase variation^2^,^3^,^16^,^17^ that increases the robustness of the population and provides opportunities to modulate transcription in response to changing environmental conditions^18^,^19^.

In some bacteria, the behaviour of certain MTases can vary markedly due to slipped-strand mispairing during DNA replication. Often occurring in homopolymer-rich regions, this slippage can cause frameshift mutations that result in truncated and usually inactive MTases^20^. Alternatively, recombination events can result in the movement of type I and III target recognition domains, which can alter the target sequence specificity of the MTase^21^,^22^,^23^,^24^,^25^. These mechanisms can yield heterogeneity in the methylomes of descendants from a single cell and can cause differential regulation of multiple genes, termed a phase-variable regulon (a.k.a. phasevarion)^20^. It has been postulated that epigenetic control of gene expression mediated by phase variation (epigenetic control of a single gene) or phasevarions (multiple genes regulated simultaneously) allows an essentially clonal population to adopt multiple distinct phenotypes^26^. Such heterogeneity can facilitate adaptation to diverse environmental niches, including complex host environments and the presence of antibiotics, as has been reported in several studies^20^,^27^,^28^,^29^,^30^.

Genomic analyses suggest that some form of DNA methylation is present in nearly all bacteria, as putative DNA MTases have been found in 94% of >3,300 sequenced bacterial genomes^5^. Given the large number of MTase target sites in bacterial genomes and the growing evidence suggesting complex regulatory roles of methylation by both R-M and orphan MTases^2^,^3^, the potential scope for exploring the diversity of bacterial methylation and methylation-mediated gene regulation is vast. However, the precise sequence targets and biological roles of most MTases remain largely unknown. While recent progress in bisulfite sequencing facilitates the accurate detection of 5mC methylation^31^, there has not been a high-throughput genome-wide sequencing methodology for efficiently detecting 6mA and 4mC methylation in bacteria.

Single molecule, real-time (SMRT) DNA sequencing technology^32^ enables the detection of nearly 20 different types of chemical modifications to DNA, including all three major types of DNA methylation in bacteria (6mA, 4mC and 5mC), although the reduced signal-to-noise ratio with 5mC makes detection of such events challenging. SMRT sequencing molecules consist of a double-stranded native DNA fragment that has been circularized by ligating hairpin adapters to each end. During sequencing, a DNA polymerase proceeds around the circularized DNA template multiple times, the exact number depending on fragment size and polymerase performance. The sequencing instrument monitors not only the pulse fluorescence associated with each incorporated nucleotide, but also the time between the incorporation events, termed the inter-pulse duration (IPD). Variation in IPDs (referred to as kinetic variation) is highly correlated with the presence of modifications within the DNA template^33^,^34^,^35^, including 6mA, 4mC, 8-oxoguanine and so on.

Many studies have used SMRT sequencing to conduct *de novo* identification of MTase target motifs, revealing surprising diversity in motifs among bacterial species and even among closely related strains^35^,^36^,^37^,^38^,^39^,^40^. However, the heterogeneities within bacterial populations cultured under different conditions have not been thoroughly explored. This is primarily limited by the current SMRT sequencing-based bacterial methylome analysis protocol, which relies on assessments of aggregate IPD values at each genomic position across populations of cells^33^,^34^,^35^,^41^; the individual reads (each from a single DNA molecule) are aligned to the same genomic region and then statistically modelled as an ensemble (Fig. 1a,b). This approach enhances the statistical power for methylation detection at single-nucleotide resolution, but fundamentally limits the ability to resolve epigenetic heterogeneity within the sample. Although progress beyond traditional population-level analysis has recently been reported^38^,^42^, new methods are needed to provide improved resolution on complex methylomes.

Here, we present SMALR, a novel framework for single molecule-level detection and phasing of bacterial DNA methylation using SMRT sequencing data. This approach relies on two complementary methods that use molecule-specific IPD information to infer methylation states at single-molecule resolution (Fig. 1c,d). We demonstrate the effectiveness of SMALR through comprehensive and quantitative characterization of seven bacterial methylomes and by identifying distinct types of heterogeneity in these methylomes. The enhanced resolution provided by SMALR allows *de novo* detection of bacterial epigenetic heterogeneity, and should broaden our understanding of the diverse roles of methylation in modulating bacterial physiology. SMALR is freely available through GitHub.

## Results

### Single molecule-level methylation detection and phasing

**Figure 1:**
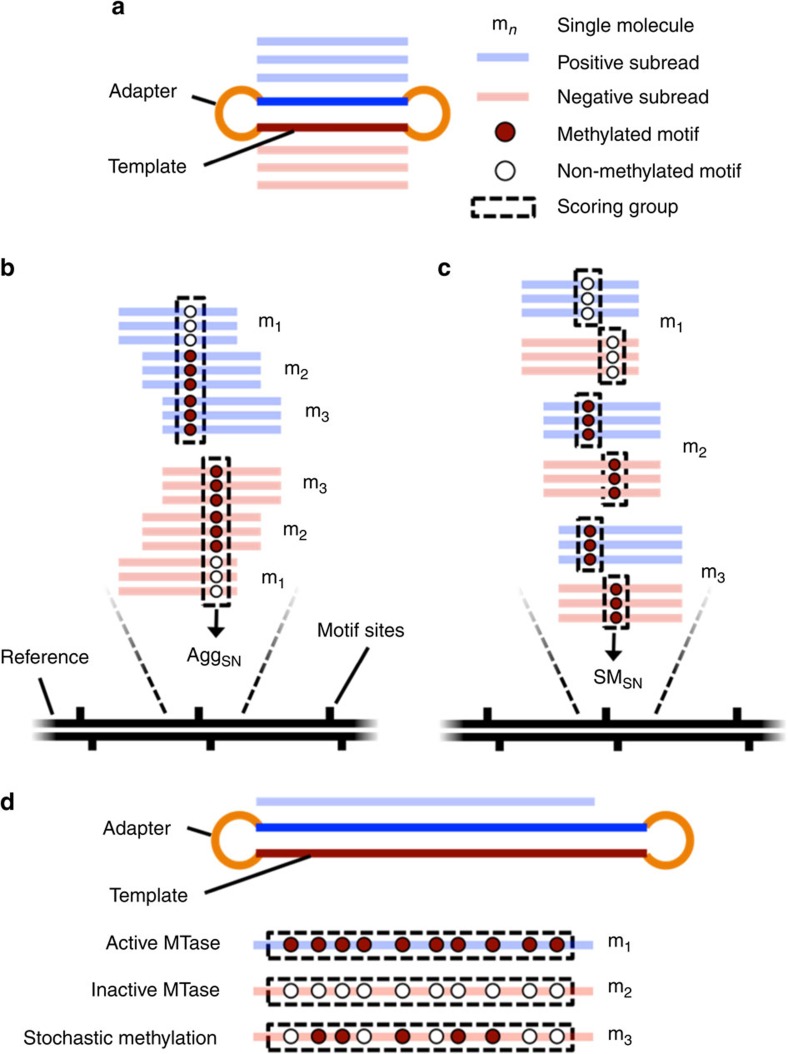
SMALR methods for methylation detection in SMRT reads. Schematic illustrating the general approaches of both the existing and two proposed SMALR methods for detecting DNA methylation in SMRT sequencing reads. (**a**) A single SMRT sequencing molecule (short DNA insert+adapters) and the subreads that are produced during sequencing. (**b**) The existing methylation detection method is based on a molecule-aggregated, single-nucleotide (Agg_SN_) score. For a given strand and genomic position, the IPD values from all the subreads aligning to that strand and position are aggregated together across all molecules to infer the presence of a consensus methylated base. (**c**) The proposed single molecule, single nucleotide (SM_SN_) method for detecting DNA methylation relies instead on separate consideration of subreads from different molecules. The SM_SN_ scores are calculated for each molecule, strand and genomic position. (**d**) A single SMRT sequencing molecule (long DNA insert+adapters) with a single long subread and the proposed single molecule, pooled (SM_P_) approach for assessing MTase activity. This approach pools together IPD values from multiple motif sites along the length of a single long subread.

We first present the rationale and description of the two complementary methods within SMALR for (i) single molecule, single nucleotide, strand-specific detection of methylation events and (ii) single molecule-level epigenetic phasing analysis. Our strategy revolves around the interrogation of circular consensus sequence data generated from short-insert SMRT sequencing libraries (∼250 bp) and continuous long-read data generated from long-insert SMRT sequencing libraries^32^,^33^. Using short insert templates, we achieved average read lengths between 4,735 and 9,023 bp (Supplementary Table 1; Methods), although recent releases of the sequencing chemistry kits promise significantly longer-read lengths (12,000 bp on average). The multiple passes of the polymerase over the same template sequence (Fig. 1a,c) allow the calculation of a single molecule, single-nucleotide (SM_SN_) score, which is defined as the difference between the mean site/strand-specific log (natural) IPD value from a native DNA molecule and corresponding mean site/strand-specific log IPD value from whole-genome amplified (WGA) DNA molecules (Methods). This score provides the statistical power to detect shifts in kinetic variation at a specific nucleotide position on a given DNA molecule.

Complementing the short-insert libraries are the long-insert libraries (our data generated in early 2014 achieved mean insert sizes of 3,357–7,304 bp and mean read lengths of 4,957–7,965 bp, although a mean read length of 12,000 bp can now be achieved; Supplementary Table 1; Methods). Most of the reads generated from this protocol represent long stretches of contiguous DNA (as opposed to the circular consensus of shorter libraries). The long library sequencing protocol has been applied to *de novo* bacterial genome assembly because of its ability to resolve genomic regions with complex repeated elements^43^,^44^,^45^. Here, we instead leverage the continuous long reads for methylation phasing at single-molecule resolution. The methylated motifs profiled in this study are represented in their genomes between 210 and 42,186 times (Supplementary Table 2), a number largely dependent on both the motif complexity and evolutionary selection. As such, a motif may be represented many times in a single long read, providing multiple data points revealing the kinetic variation for the motif. By pooling these kinetic variation statistics for a single read, it becomes possible to not only infer whether the cell that provided that DNA fragment contained MTase activity targeting that motif, but also to quantitatively estimate the processivity of the MTase (Fig. 1d). We therefore created the single molecule, pooled (SM_P_) score, which is defined as the difference between the mean log IPD value from all motif sites in a single native subread and the mean log IPD value from those same motif sites in WGA DNA molecules (Methods). Although this estimation procedure is more powerful for shorter methylation motifs than for the longer motifs that occur less frequently, the relatively longer-read lengths (reads up to 60,000 bp are now possible with recent updates to the SMRT chemistry kit) provide an opportunity to estimate the SM_P_ scores accurately for longer motifs.

### Sensitivity and specificity of SM_SN_ detections

**Figure 2:**
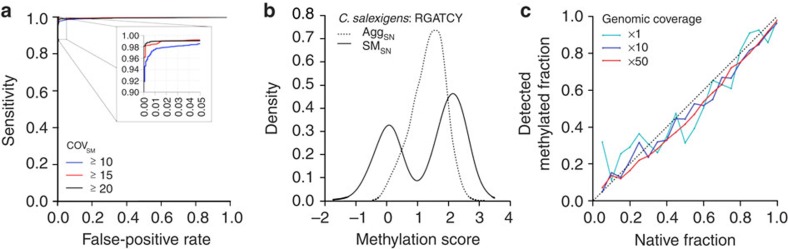
Performance of SM_SN_ level detection of DNA methylation. Multiple metrics showing the performance of the proposed single molecule, single nucleotide (SM_SN_) detection method. (**a**) Performance of the approach for detecting 6mA modifications in the 5′-CTGCAG motif of *E. coli* C227 using three thresholds for minimum single-molecule coverage (cov_SM_). (**b**) Distribution of the aggregate, single nucleotide (Agg_SN_) and SM_SN_ methylation scores for the partially non-methylated 5′-RGATCY motif in *C. salexigens*. The bimodal distribution of the SM_SN_ scores enables the accurate and objective estimation of this fraction. (**c**) Accuracy of SM_SN_-enabled estimations of the methylated fraction (using cov_SM_≥10) for the 5′-CTGCAG motif of *E. coli* C227 at various levels of genomic-sequencing coverage.

An accurate mean log IPD for each nucleotide in a single molecule permits confident estimation of the IPD ratio. The accuracy of the mean log IPD increases as the number of subreads for each molecule increases^33^,^34^, as each subread provides an independent estimate of the IPD. For a given read length, the number of subreads (that is, single-molecule coverage, cov_SM_) is negatively correlated with the insert size of the sequencing library. To quantify the sensitivity and specificity for SM_SN_ detection, we analysed methylated 5′-CTGCAG sites in a native *E. coli* O104:H4 C227-11 strain^35^ (referred to as C227), and a matching WGA sample that is free of methylation (Fig. 2a; Methods). As expected, the sensitivity of the method increased with increasing cov_SM_. For the SM_SN_ approach, cov_SM_≥15 enables detection of 6 mA with a sensitivity of 98.5% and a specificity of 99.5%. Although smaller library sizes can provide higher values of cov_SM_, a minimum library size of 150–200 bp is recommended to avoid loss of genomic DNA during library construction and to facilitate removal of adapter-dimer constructs during purification.

Estimating the sensitivity and specificity of detecting 6mA at single-molecule resolution for other 6 mA motifs, we found the results to be comparable to 5′-CTGCAG in strain C227 (Supplementary Fig. 1a,b). We found that 4mC also can be accurately detected at single-molecule resolution, albeit with slightly lower sensitivity and specificity (Supplementary Fig. 1c). We did not attempt similar analysis of 5mC motifs due to the significantly lower signal-to-noise ratio^24^,^38^, even after conversion of 5mC to 5hmC with Tet enzymes^46^. The remainder of this report will focus solely on the characterization of 6 mA due to both its prevalence in bacteria and the substantial body of evidence supporting its important regulatory potential^12^,^22^,^23^; however, all of the analyses detailed also are applicable to 4mC.

### SM_SN_ scores for estimating global methylation heterogeneity

The methylome of *Chromohalobacter salexigens* characterized in a recent study was found to contain a substantial number of non-methylated 5′-RGATCY sites^37^. In particular, 23.5% of the motif sites were predicted to be non-methylated based on the standard population-level analysis, which we replicated using molecule-aggregated, single-nucleotide (Agg_SN_) scores (Fig. 2b; Methods). As shown, the distribution of Agg_SN_ scores does not show clear separation between methylated and non-methylated 5′-RGATCY sites, indicating that quantifying the methylated fraction of 5′-RGATCY sites in the genome with Agg_SN_ scores relies on a subjective and *ad hoc* threshold. In contrast, there is clear bimodality in the distribution of SM_SN_ scores, where the components centred near SM_SN_≈0 and SM_SN_≈2 represent the non-methylated and methylated fractions, respectively (Fig. 2b). A distribution of SM_SN_ centred at zero correspond to motif sites that are not methylated because the IPDs do not differ between native and WGA DNAs; in contrast, a distribution of SM_SN_ centred near two corresponds to motif sites that are methylated (specific to 6 mA; consistently observed across multiple bacterial species and motifs as shown in Supplementary Figs 3-8). This bimodality allows the percentage of methylated motif sites to be estimated at 60.4% using a standard expectation maximization (EM) algorithm^47^ (Methods) without need for a subjective input threshold.

### SM_SN_-based quantification at low sequencing coverage

As the SM_SN_ scores depend on cov_SM_ rather than the genomic-sequencing coverage, we conducted an *in silico* experiment to test the accuracy of SM_SN_-based estimates of global methylated fraction at varying levels of genomic coverage. By mixing native and WGA-sequencing molecules at varying proportions and by gradually reducing the total number of molecules, we found that the SM_SN_-based estimates of the global methylated fraction are stable even when the genomic sequencing coverage is as low as × 1 (Fig. 2c; Methods). This finding could have implications for the characterization of *in vivo* isolates, for which low sequencing coverage due to limited DNA input is often a challenge.

### Global methylation heterogeneity in six bacteria

**Figure 3:**
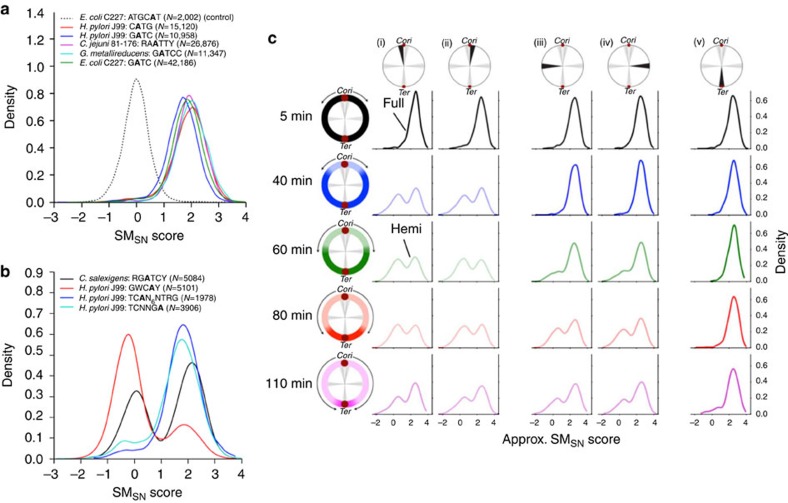
SM_SN_ score distributions reveal epigenetic heterogeneity. (**a**) Single molecule, single nucleotide (SM_SN_) score distributions for multiple bacterium-motif pairs (and the genome-wide motif count, *N*, of each motif) that exhibit near complete methylation, along with a non-methylated motif for comparison. (**b**) SM_SN_ score distributions for multiple bacterium-motif pairs that display significant non-methylated fractions. The *H. pylori* J99 motifs show minor variation in the SM_SN_ associated with each peak due to subtle differences in the chemistry version used for SMRT sequencing of the native and WGA samples. (**c**) SM_SN_ interrogation of 5′-GANTC methylation at five genomic positions (columns) in a synchronized *C. crescentus* culture during a single round of DNA replication. Five time points (minutes post-synchronization; rows) provide snapshots of the bidirectional progression of the replication forks from the origin of replication (*Cori*) to the terminus (*Ter*). Grey wedges in the chromosome schematics show the 200-kb genomic regions where the SM_SN_ scores are queried for each time point. Two regions are on either side of the *Cori*: (i) *Cori* - 0.1 Mbp and (ii) *Cori*+0.1 Mbp. Another two are halfway between *Cori* and *Ter*: (iii) *Cori* - 1 Mbp and (iv) *Cori*+1 Mbp. The final region covers the terminus: (v) *Ter*. Light (hemimethylated) to dark (fully methylated) colour shading in the schematic illustrates the approximate position of the replication fork at each time point. The bimodal distributions of approximate SM_SN_ scores (Methods) reveal the progressive hemi-methylation of 5′-GANTC sites following the passage of the replication forks. Hemimethylated sites cannot transition back to full methylation until the MTase gene, *ccrM*, is transcribed, which does not occur until late in the replication process.

We applied the SM_SN_ analysis to six bacterial methylomes that were recently sequenced^35^,^37^,^38^ or were specifically sequenced for this study (Supplementary Table 1). We first detected methylation motifs based on existing methods^24^,^35^,^37^ and divided them into two groups based on the global distribution of SM_SN_ scores. In the first group (Fig. 3a), most (>95%) motif sites were methylated, with only a small proportion non-methylated, likely due to competitive binding between the MTases and other DNA-binding proteins such as transcription factors^2^,^3^,^11^,^13^,^14^. In the second group (Fig. 3b), a substantial (>5%) percentage of motif sites were non-methylated, suggesting the existence of alternative mechanisms that drive methylome heterogeneity. After analysing the SM_SN_ scores for all bacterium-motif pairs (Supplementary Figs 3-8), we observed that while most motifs belong to the first group and do not show extensive non-methylation, the second group includes the 5′-RGATCY motif of *C. salexigens* and three motifs from *Helicobacter pylori* J99. Most intriguingly, the *H. pylori* motif 5′-GWCAY shows a very high (75.3%) percentage of non-methylated sites, which we subsequently investigated using the SM_P_ analysis (see below).

### SM_SN_ score distributions show regional methylation dynamics

The *Caulobacter crescentus* genome encodes a methyltransferase (CcrM) targeting 5′-GANTC sites^48^. The corresponding gene, *ccrM*, is only expressed at late stages of the cell cycle. Consequently, fully methylated 5′-GANTC sites transition to hemi-methylated as the replication fork proceeds from the origin of replication (*Cori*) to the terminus (*Ter)*^38^. Recently, Kozdon *et al.* 38 used SMRT sequencing to study such transitions at five time points spanning a single, synchronized round of the cell cycle. This allowed us to conduct an approximate SM_SN_ analysis (approximate due to lack of WGA sequencing in the original data; Methods) to test the use of SM_SN_ for detecting regional methylation heterogeneity, focusing specifically on five distinct 200-kilobase regions in each of five time points (Fig. 3c).

As the replication fork proceeds from the *Cori* to the *Ter* in the five time points, the SM_SN_ score distributions reveal an increasing fraction of the genome that has been converted from fully methylated to hemi-methylated 5′-GANTC sites. At the first time point (5 min post-synchronization), the fully methylated state of the cells is shown by the single mode in approximate SM_SN_ scores (Methods) in all five genomic regions. As the cells begin to differentiate (40 min), there are bimodal SM_SN_ scores in regions (i) and (ii) that are closest to the *Cori,* reflecting the passage of the replication fork through those regions. The next two time points reveal the transition of the single-mode approximate SM_SN_ distributions in regions (iii) and (iv) to clear bimodal distributions, reflecting the passage of the replication fork through those regions. The unequal bimodal distribution after 80 min in regions (iii) and (iv) indicates that the molecules from that region have not all been converted from fully to hemi-methylated, likely due to stochastic variance in the position of the replication fork 80 min post-synchronization. The final time point reveals a genome that has almost completely converted to a hemi-methylated state, with the exception of the region immediately surrounding the *Ter* (v), which shows only a small amount of hemi-methylation. Analysis at this time of regions (i)-(iv) indicates that these sites have already begun to transition from equal bimodality (universal hemi-methylation) towards their starting single-mode distributions (universal full methylation). This likely is also due to imperfect synchronization, allowing some replication forks to progress past the *ccrM* gene at the LPD time point, thus activating transcription and beginning the process of re-methylating all the 5′-GANTC sites that had been rendered hemi-methylated by passage of the replication fork.

### SM_P_ identifies distinct types of epigenetic heterogeneity

We considered three potential explanations for the heterogeneous methylation observed in select bacterium-motif pairs (Fig. 3b). First, we hypothesized that methylation motifs, especially degenerate motifs, may be inaccurately annotated; the observed heterogeneity would simply be due to a mixture of truly methylated motifs and motifs falsely identified by the motif enrichment algorithm. For example, the heterogeneous methylation of the 5′-RGATCY motif in *C. salexigens* (Fig. 2b) may result from only three of the four explicit specifications (A/G)GATC(C/T) being truly methylated. However, we can reject this hypothesis as the SM_SN_ score distributions of the four explicit specifications showed similar heterogeneity (Supplementary Fig. 9).

A second possibility is that the MTase is stochastically methylating only a fraction of its recognition motif sites in each cell, in which case the SM_SN_-based estimates of methylated fraction reflect a universally active MTase, albeit one without the ability to methylate all of its target motifs. In that case, we would expect the methylated motif sites to be interspersed with non-methylated sites throughout a single copy of the genome.

A third potential cause of heterogeneity in SM_SN_ scores is phase variation of the MTase responsible for targeting the motif. Phase-variable MTases have been well-documented in a variety of bacteria, in which stochastic mutations in homopolymers or other simple sequence repeats in the MTase can induce changes in the transcribed product that either activate/deactivate the MTase or modify its target specificity^20^,^22^,^24^. Either of these two switching modes can induce cell-wide methylation patterns that might differ between neighbouring cells in a single population. In this scenario, the methylated fraction estimated by SM_SN_ scores reflects the fraction of cells with an active MTase targeting the motif.

We can differentiate between these latter two hypotheses (intra-cellular stochastic methylation versus stochastic phase-variable MTase with different phases in different cells) by using the SM_P_ method to phase epigenetic information across the full length of each read (that is, methylation co-occurrence on a single molecule). If some molecules within a sample are methylated at all sites while others are completely non-methylated, this supports the existence of a stochastic phase-variable MTase. In contrast, a mixture of methylated and non-methylated motif sites on a single read suggests intracellular stochastic methylation as the source of heterogeneity in the SM_SN_ scores.

### Direct detection of phasevarion in single *H. pylori* colonies

Phase-variable genes regulated by length variation of homopolymeric tracts have been well-documented in *H. pylori*^20^,^22^,^49^,^50^,^51^,^52^. Several putative MTases contain inactivating frameshift mutations that prevent the transcription of an active protein. By experimentally correcting frameshifts in several MTases and SMRT sequencing the mutated populations, Krebes *et al.* were able to detect methylation (using aggregation-based methods) by the re-activated MTases and identify their target motifs^24^. However, because most *H. pylori* isolates are likely not clonal and significant homopolymer length variation has been observed in MTases between closely related strains of *H. pylori*^49^, it is possible that active copies of the phase-variable MTases are already present in the wild-type isolates, but at levels too low to detect using the aggregation-based method. This problem provided an opportunity to use the SM_P_ method for direct detection in wild-type isolates of minor subpopulations with active and inactive MTases.

**Figure 4:**
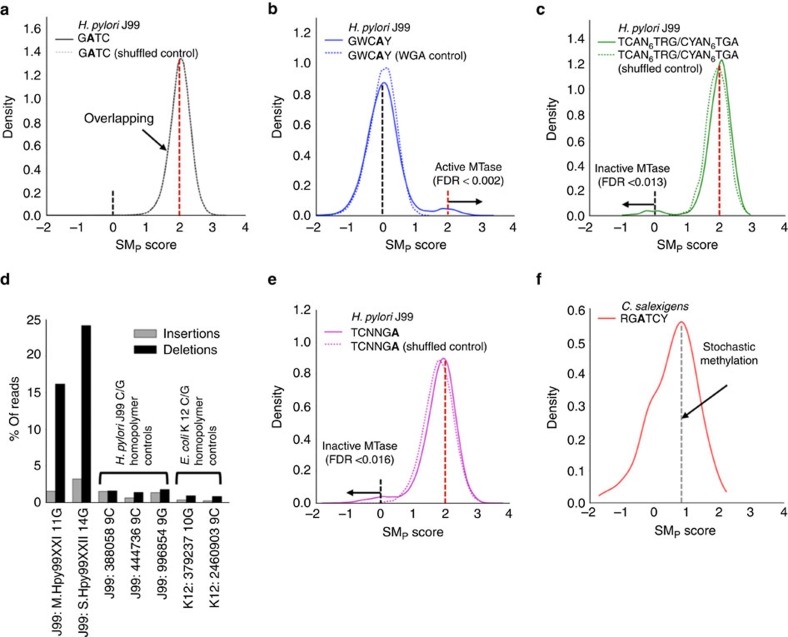
SM_P_ score distributions reveal distinct types of epigenetic heterogeneity. (**a**) Single molecule, pooled (SM_P_) distribution for *H. pylori* J99 motif 5′-GATC and its corresponding IPD-shuffled control. The identical unimodal distributions suggest a fully active MTase (as expected). (**b**) SM_P_ distributions for *H. pylori* J99 motif 5′-GWCAY and its corresponding WGA control. The major peak around SM_P_≈0 and minor peak around SM_P_≈2 suggests that the mostly inactive MTase targeting 5′-GWCAY, M.Hpy99XXI, is methylating 5′-GWCAY in a small fraction of cells. Methylated molecules with SM_P_ scores>2 have an FDR<0.2%. (**c**) SM_P_ distributions of 5′-TCAN_6_TRG/5′-CYAN_6_TGA in *H. pylori* J99 and its corresponding IPD-shuffled control. The major peak around SM_P_≈2 and minor peak around SM_P_≈0 indicates that the normally active MTase, Hpy99XXII, is inactive in a small fraction of cells. Non-methylated molecules with SM_P_ scores<0 have an FDR<1.3%. (**d**) High-accuracy sequencing with Illumina MiSeq and read-level analysis of insertion/deletion calls shows significant variation in the lengths of two specific homopolymers in the coding sequences of M.Hpy99XXI and S.Hpy99XXII. The high percentage of deletions in these two genes stands apart from the deletion rates found in five other C/G homopolymers from *H. pylori* J99 and *E. coli* K12, suggesting that this is not simply due to lower sequencing accuracy in homopolymer regions. (**e**) SM_P_ distributions of 5′-TCNNGA in *H. pylori* J99 and its corresponding IPD-shuffled control. The SM_P_ scores suggest a MTases behaviour similar to that of Hpy99XXII. Non-methylated molecules with SM_P_ scores<0 have an FDR<1.6%. (**f**) SM_P_ distribution for the *C. salexigens* motif 5′-RGATCY. The major peak near SM_P_≈0.9 indicates that the IPDs sampled for each molecule reflect a mixture of both non-methylated (IPD≈0) and methylated (IPD≈2) motif sites, suggesting stochastic methylation as the primary source of epigenetic heterogeneity for this motif.

We first tested the SM_P_ method on an *H. pylori* J99 isolate that was sequenced using libraries with long (∼20 kb) DNA inserts. We targeted the 4-mer 5′-GATC motif, where >95% of sites are expected to be methylated based on its SM_SN_ distribution (Fig. 3a), and calculated a SM_P_ score for each long read containing at least 10 GATC sites. The distribution of SM_P_ scores for 5′-GATC was compared with a control distribution of SM_P_ scores calculated after randomly shuffling IPD values between molecules (Fig. 4a). No bimodality is present in the 5′-GATC SM_P_ distribution and it is nearly identical to the IPD-shuffled SM_P_ distribution, suggesting that the MTase responsible for targeting the 5′-GATC motif in *H. pylori* J99 (M.Hpy99VI) is constitutively active. Through false-discovery rate (FDR) estimation (Methods), we found that only 0.07% of the molecules with at least 10 5′-GATC sites had evidence of non-methylation (maximum FDR=1%). This level of non-methylation is consistent with several other motifs for which we expect to observe near-universal methylation activity (5′-CATG, 5′-GANTC and 5′-GAGG), suggesting that the small number of non-methylated molecules may have originated from transiently hemi-methylated regions directly behind the DNA replication fork. Furthermore, phase variation of M.Hpy99VI was considered unlikely as no significant sequence variation was observed in its coding sequence (Supplementary Fig. 10).

Next, we applied the same analysis to a motif, 5′-GWCAY, targeted by a known phase-variable MTase in *H. pylori* J99. The modification (M) subunit of the type III R-M system Hpy99XXI contains a 10-12G homopolymer locus, the exact length of which determines whether transcription downstream of the locus is in-frame (active MTase) or out-of-frame (inactive MTase). Methylation of the 5′-GWCAY motif is normally undetectable in *H. pylori* J99 using aggregation-based methods, suggesting that the majority of M.Hpy99XXI proteins are inactive^24^. However, the SM_P_ distribution shows (Fig. 4b) a subpopulation of molecules with SM_P_≈2, indicative of a subpopulation of cells containing the active form of M.Hpy99XXI. Illumina MiSeq sequencing of the same DNA supported this mechanism, revealing a substantial length variation in the *M.Hpy99XXI* homopolymer (Supplementary Fig. 11; Methods).

Hpy99XXII is another R-M system in *H. pylori* J99 known to contain phase-variable components. In contrast to the Hpy99XXI system, the phase variation in this system is due to homopolymer length variation within the specificity (S) subunit that targets the 5′-TCAN_6_TRG/5′-CYAN_6_TGA motif. The SM_P_ scores for this motif show that, although the majority of molecules have scores suggesting active methylation, there is a subpopulation of molecules with SM_P_≈0 (Fig. 4c), indicative of cells within the culture lacking a functional Hpy99XXII system. MiSeq sequencing confirmed the presence of significant *S.Hpy99XXII* homopolymer length variation (Supplementary Fig. 12).

On most platforms, there is reduced sequencing accuracy of homopolymeric regions, especially poly-C/G tracts. To ensure that the observed insertions and deletions were not due to sequencing errors, we searched for length variation in other C/G homopolymers in the *H. pylori* J99 and *E. coli* K12 genomes that are not linked to phase variation (Fig. 4d). The number of read-level deletions (Methods) observed in the *M.Hpy99XXI* and *S.Hpy99XXII* genes is markedly higher (>15%) than those observed (<3%) in the control C/G homopolymers, indicating that true length variation, rather than sequencing error, is driving the considerable read-level variation seen in these two homopolymers.

The *H. pylori* J99 strain used in this study has been passaged many times over the 15 years since its original isolation^53^. While phase variation refers to rapid state switching in response to the environment, it is also possible that, over the course of many passages, stable subpopulations have diverged within the isolate, each containing distinct and fixed homopolymer lengths. To investigate this possibility, we isolated five single colonies (HPXZ1383-1387; Methods) from the original isolate for Illumina sequencing and subjected the reads to analysis of read-level variation (Methods). Even in these clonal isolates, significant length variation was observed in the homopolymers within the *M.Hpy99XXI* and *S.Hpy99XXII* genes (Supplementary Fig. 13). This indicates that homopolymer length variation at these loci develops rapidly, generating heterogeneity in MTase activity in the descendants from a single cell. It is also worth noting that one of the five colonies revealed a large percentage of deletions in M.Hpy99XXI (that is, mostly 10G homopolymer rather than 11G), suggesting that this single colony is likely derived from a parent cell with a 10G homopolymer.

Interestingly, although a similar distribution of SM_P_ scores was observed for both the 5′-TCAN_6_TRG/5′-CYAN_6_TGA (Fig. 4c) and 5′-TCNNGA (Fig. 4e) motifs, no sequence variation was observed in or near the coding region of the genes involved in the Hpy99XVIII R-M system that methylates 5′-TCNNGA (Supplementary Fig. 14). This finding suggests that regulatory elements outside the coding region (genetic or epigenetic) may be responsible for the observed fraction of 5′-TCNNGA non-methylated molecules. Analysis of SM_P_ scores for other methylated motifs in *H. pylori* J99 did not reveal significant bimodality, suggesting full MTase activity (Supplementary Fig. 15).

### Gene expression in M.Hpy99XXI and S.Hpy99XXII mutants

To assess the effect of a genome-wide absence of methylated 5′-GWCAY and 5′-TCAN_6_TRG/5′-CYAN_6_TGA motifs, we compared the transcriptomes of the five clonal wild-type J99 isolates and the two mutant strains lacking *M.Hpy99XXI* and *M.Hpy99XXII* (HPXZ1401 and HPXZ1398, respectively; Methods). In the strain lacking 5′-GWCAY methylation (HPXZ1401; Supplementary Table 5), 38 genes were significantly differentially expressed, while the strain lacking 5′-TCAN_6_TRG/5′-CYAN_6_TGA methylation (HPXZ1398; Supplementary Table 6) contained 41 significantly differentially expressed genes (*P*<0.001; an adapted Fisher's exact test (ref 70); FDR<0.05). The most striking changes in HPXZ1401 were the upregulation of *flgE*, which encodes the flagellar hook protein FlgE, and two other flagellum-related genes, *flgB* and *flaG* (Supplementary Table 5). Flagella are essential for *Helicobacter* motility and are subject to phase variation via reversible length variation in a short homopolymeric sequence repeat in the *fliP* gene^54^. The upregulation of three flagella-related genes in HPXZ1401 may suggest that other novel mechanisms are involved in *H. pylori* motility switching. Two other significantly upregulated genes, *groEL* and *groES*, which reside in the same operon based on *in silico* prediction^55^ and transcription start site mapping in another strain^56^, showed 2.0- and 5.4-fold enrichment, respectively, of 5′-GWCAY motif sites (Supplementary Fig. 16) compared with the average genome-wide frequency. Such chaperone-related genes play important roles in bacterial stress responses to the host environment^57^. The expression changes in HPXZ1398 also include the flagellar hook-encoding gene, *flgE*, and several genes related to DNA processing and metabolism (Supplementary Table 6). These results demonstrate that genome-wide alterations of methylation patterns can significantly impact gene expression, although the specific mechanisms by which methylation regulates the expression of these genes are unknown.

### Intracellular stochastic methylation in *C. salexigens*

In contrast to 5′-GWCAY, 5′-TCAN_6_TRG/5′-CYAN_6_TGA and 5′-TCNNGA in *H. pylori* J99, the SM_P_ scores of the 5′-RGATCY motif in *C. salexigens* do not support the existence of phase-variable MTase activity (Fig. 4f). Instead of a peak near SM_P_≈2, there is a peak near SM_P_≈0.9, indicating that the IPD values used to calculate SM_P_ scores for each molecule reflect a mixture of both non-methylated and methylated motif sites. This observation combined with a lack of detected sequence variation in the coding sequence of the 5′-RGATCY-targeting *M.CsaI* gene (Supplementary Fig. 17) and that M.CsaI is an orphan MTase without a corresponding RE^37^ indicates that intracellular stochastic methylation by M.CsaI is the likely mechanism driving the observed heterogeneity in SM_SN_ scores (Fig. 3b). Therefore, M.CsaI in *C. salexigens* may have stochastic methylation activity (∼60%) in each cell, constituting an alternate form of epigenetic heterogeneity in a bacterial population.

## Discussion

Here, we present SMALR, the first systematic framework for single molecule-level characterization of epigenetic heterogeneity in bacterial methylomes using SMRT sequencing. We demonstrate the enhanced resolving power of our approach in analyses of seven methylomes that show distinct types of epigenetic heterogeneity in bacterial populations. The short library-based SM_SN_ method enables accurate estimation of the fractions of methylated and non-methylated motif sites without the use of subjective thresholds, even at low levels of genomic sequence coverage. Furthermore, the robust separation between methylated and non-methylated sites based on SM_SN_ scores at low levels of genomic sequence coverage suggests the possibility of reference-free, *de novo* discovery of methylation motifs from SMRT sequencing data at a much lower coverage than is currently required. The long library-based SM_P_ method uses long reads to assess the co-occurrence patterns of methylated motif sites at single-molecule resolution. By surveying MTase activity at the single molecule-level, the SM_P_ scores can reveal small fractions of cells in a bacterial population that contain active or inactive MTases, and quantitatively estimate the processivity of an MTase of interest.

The existing, consensus-based methods for methylation detection cannot provide the resolution necessary to survey complex MTase activity. The application of SMALR and its integration with other single molecule- or single cell-level data, such as RNA and protein expression will enable a more detailed understanding of the functions of DNA methylations in bacterial physiology.

DNA methylation in promoter regions of bacteria has been extensively linked to the regulation of nearby genes^2^,^3^,^12^,^58^, but there is growing appreciation for the potential roles of methylation in the gene body^31^,^59^,^60^. More generally, the importance of epigenetic regulation in bacteria is emphasized in a recent study that proposed an epigenetics-driven adaptive evolution model^21^. In this model, heterogeneous methylomes may drive unique patterns of gene expression and cellular phenotypes, thereby serving as units of natural selection. The methods presented here make it possible to characterize the epigenetic modes that induce transcriptional diversity, analogous to the many known genetic modes^17^,^26^,^49^,^54^,^61^.

While SMRT sequencing enables *de novo* detection of a wide variety of DNA modifications, it also poses challenges unique to third-generation sequencing technology. Specifically, detection of DNA modifications from SMRT reads fundamentally depends on the number of repeated observations for each single molecule. For a given read length, there is a resulting tradeoff between library size and accuracy in single molecule, single nucleotide-level methylation detection. The methods proposed here provide examples of effectively leveraging the unique features of SMRT sequencing using a combination of short- and long-library designs. This general methodology can be modified to accommodate forthcoming third-generation real-time sequencing techniques^62^.

While the current study uses SM_SN_ scores from a single site to detect DNA methylation, previous work has demonstrated that some DNA modifications (DNA methylation or damage) can create kinetic signatures that extend into surrounding bases^34^. Although beyond the scope of the current work, the single molecule-level approaches presented here can be expanded to include more sophisticated algorithms required for detection of these modification types.

Finally, while the current study focused on cultures of single bacterial strains, the single-molecule resolution methods proposed here can also be applied to mixed populations of bacteria. Such samples could include diverse clinical isolates, including samples that contain a high abundance pathogen or even diverse microbiome samples. The methods are also applicable outside the bacterial kingdom, such as in the analysis of human mitochondrial DNA or DNA viruses, both of which present significant genetic and epigenetic heterogeneity.

## Methods

### Code availability

SMALR is implemented in a stand-alone software package (written in Python) and available at https://github.com/fanglab.

### Bacterial strains and culture conditions

The *E. coli* C227 sample was isolated from a 64-year-old woman from Hamburg, Germany, who was hospitalized in Copenhagen, Denmark after presenting with bloody diarrhoea. Rasko *et al.*^44^ isolated DNA using a Qiagen DNEasy Blood and Tissue Kit as per manufacturer's instructions. The isolate was grown overnight in standard LB broth and the extraction was performed according to the kit instructions using 1 ml overnight culture per reaction and treating with proteinase K for 2 h. The DNA was eluted in 100 μl AE buffer per column.

*H. pylori* J99 was originally isolated in Nashville Tennessee from an American patient with a duodenal ulcer^63^ and sequenced in 1999 (ref. 53). Cells (50 μl) of frozen *H. pylori* J99 stock from the original isolating lab were first spotted on trypticase soy agar (TSA) plates with 5% sheep blood (TSA, BBL Microbiology Systems, Cockeysville, MD) at 37 °C with 5% CO_2_ for 48-h incubation, then were spread on new TSA plates in the same conditions^64^. After 24-h incubation, *H. pylori* cells were collected in 1.0 ml phosphate-buffered saline (PBS, pH 7.4) and centrifuged at 800 g for 5 min. Bacterial genomic DNA was prepared using the Wizard Genomic DNA purification kit according to manufacturer's instructions (Promega, Madison WI). DNA concentration was measured by Nanadrop 1,000 spectrophotometer (Thermo Scientific, Rockford, IL).

DNA for *Chromohalobacter salexigens* strain 1H11 was ordered from DSM ( http://www.dsmz.de/catalogues/details/culture/DSM-3043.html).

Kozdon *et al.*^38^ grew *C. crescentus* cells at 28 °C in M2-glucose minimal media (M2G) and performed small-scale synchrony. To have sufficient volume to remove large aliquots at each time point, swarmer cells were pooled from 16 small-scale synchronies. Cells were allowed to proceed synchronously through the cell cycle at 28 °C in M2G. Genomic DNA was isolated using the Gentra Puregene Yeast/Bacteria Kit (Qiagen). Cells were removed from synchronous cultures growing in M2G. Genomic DNA was isolated from 4 ml of cells at each time point. The protocol provided with the kit was followed with a few modifications: 4 μl RNaseA was used instead of 1.5 μl, and after protein precipitation the samples were left on ice for 15 min and pelleted for 5 min at 4 °C. The DNA pellet was allowed to dry for 10 min and then resuspended in 25 μl 10 mM Tris, pH 8.0. Samples were incubated at 65 °C for 1 h and at room temperature overnight^38^.

Murray *et al.*^37^ received the DNA of *G. metallireducens* from an established culture collection^65^. Murray *et al.* received *C. jejuni* 81–176 and *C. jejuni* NCTC 11168 DNAs from Stuart Thompson, Medical College of Georgia.

### SMRT sequencing

Long insert DNA library preparation and sequencing was performed according to the manufacturer's instructions. Upon completion of library construction, samples were assessed for quantity and insert size using an Agilent DNA 12,000 gel chip. Additional size selection was conducted using Sage Science Blue Pippin 0.75% agarose cassettes to enrich for library in the range of 7,000–50,000 bp. This selection is necessary to narrow the library distribution and maximize the SMRTbell sub-readlength. 11–23% of the input libraries was eluted from the agarose cassette and was available for sequencing. For all cases, this yield was sufficient to proceed to primer annealing and DNA sequencing on the Pacific Biosciences RSII machine. Primer was then annealed to the size-selected SMRTbells with the full-length libraries (80 °C for 2 min 30 followed by decreasing the temperature by 0.1°per second to 25 °C). The polymerase–template complex was then bound to the P5 (or P4) enzyme using a ratio of 10:1 polymerase to SMRTbell at 0.5 nM for 4 h at 30 °C and then held at 4 °C until ready for magbead loading, before sequencing. The magnetic bead-loading step was conducted at 4 °C for 60 min per manufacturer's guidelines. The magbead-loaded, polymerase-bound, SMRTbell libraries were placed onto the RSII machine at a sequencing concentration of 75 pM and configured for a 180 min continuous sequencing run.

For all short (250 bp) insert library preparations, similar methodology was used, except shearing was done using a Covaris microtube ultrasonication and all AMPure XP purification steps were done using a 1.8 × volume ratio. Libraries were completed without the size selection step used for the long-insert libraries. Similar procedures were followed for sequencing, except that diffusion-based loading was used instead of magbead loading.

### Filtering subreads and preprocessing SMRT reads

An initial filtering step removes all subreads with ambiguous alignments (MapQV<240), low accuracy (<80%) or short-aligned length (<100 bases). Next, because sequencing errors in the subreads are likely to introduce noise into the IPD distribution that is being used to infer methylation status, an additional filtering step removes the subread IPD values from the positions +1:−1 on either side of any errors with respect to the reference sequence. This removes a substantial number of IPD values from consideration, but those that remain are minimally impacted by sequencing errors. Subread IPD normalization corrects for any potential slowing of polymerase kinetics over the course of an entire read (which consists of many subreads) and is done by log-transforming all subread IPD values and subtracting their mean from each individual log-transformed IPD value. Finally, the first ten and last fifteen bases from each subread are removed from analysis due to the potential for bias in the IPD values near the transition between template and adapter sequences.

### SM_SN_ detection of methylation states

Considering each native molecule separately, the IPD values (post-filtering) for a given motif are grouped by their strand and mapped genomic position. Following natural log conversion of the set of IPD values (transforming exponentially distributed IPD to follow an approximately normal distribution) for the molecule/strand/position, the mean value is calculated. Contrary to the treatment of native IPD values, the WGA IPD values are aggregated across all molecules covering each strand and mapped genomic position. This aggregation is done because all molecules are expected to be free of any DNA methylations due to the amplification process^33^,^35^. The SM_SN_ score is calculated by subtracting the WGA strand/position-matched mean log IPD value from this native molecule/strand/position-specific mean log IPD value. This score approximately follows a normal distribution^34^.

### Whole-genome amplification

The Qiagen REPLI-g amplification kit was used to perform whole-genome amplification to exclude epigenetically modified bases. The method produced micrograms of DNA from 50 ng of input genomic DNA, following the manufacturer's guidelines and 10 h of amplification time at 30 °C followed by deactivation at 65 °C for 3 min.

### SM_P_ detection of MTase activity

First, considering each molecule separately, the native IPD values for a given motif that survive filtering are grouped by their strand only, irrespective of their mapped genomic positions. The natural log is taken for all values in this group and the mean calculated. Second, the strand/position-matched WGA IPD values are aggregated across all molecules, as they were with the SM_SN_ method. However, here the WGA IPD values are additionally aggregated across each motif site covered by the native molecule in question. Then, these WGA strand- and motif site-matched IPD values are natural log-converted and their mean value is subtracted from the native molecule/strand-specific mean, resulting in the SM_P_ score.

### Sensitivity and specificity of SM_SN_ methylation detection

To quantify the sensitivity and specificity for SM_SN_ detection, we applied the approach to methylated 5′-CTGCAG sites in a native *E. coli* O104:H4 C227-11 strain^35^ (C227) and a matching WGA sample in which the methylation sites were erased. In the analysis, we assumed 100% of the 5′-CTGCAG sites were methylated in the native DNA to leverage the large number of motif sites in the native bacterial genome. This is expected to provide a more robust estimation of sensitivity and specificity compared with the use of short DNA oligos that have limited diversity of expanded local sequence contexts flanking any given methylation motif site^34^,^66^. Given that 5′-CTGCAG sites are part of an active type-II R-M system, it is reasonable to assume the vast majority of sites are methylated to prevent restriction of the host DNA. However, as shown in recent studies^35^,^36^,^38^, a small number of motif sites (even those that are part of R-M systems) are sometimes found to be stably non-methylated, likely due to competitive binding between the MTase and other DNA-binding proteins or to the transient non-methylated window that follows behind the replication fork. Because the true methylated fraction of 5′-CTGCAG sites is slightly lower than 100%, the sensitivity estimated in the above analysis represents the lower bound of the actual sensitivity of the SM_SN_ approach.

### Agg_SN_ detection of methylation states

The IPD values from all native molecules were aggregated according to their strand and mapped genomic position. Similarly, the IPD values from all the whole-genome amplified (WGA) molecules were aggregated according to their strand and mapped genomic position. The Agg_SN_ score per strand/position represents the difference between the mean native log IPD value and the mean WGA log IPD value.

### Modified fraction estimates with Gaussian mixture modelling

The mixture module of the Python package *PyMix* was used to run the EM algorithm^67^ for Gaussian mixture estimation.

To evaluate the ability of EM with a Gaussian mixture model to estimate the modified fraction, we applied the algorithm to distributions of SM_SN_ scores that were generated from *in silico* mixing of WGA and native SMRT sequencing molecules at known proportions. 100,000 total molecules sequenced from *E. coli* C227 were used for each specific mixture fraction, with the native fraction of molecules ranging from 5,000 (5%) to 100,000 (100%). The SM_SN_-based methylated fraction for the motif 5′-CTGCAG was analysed with the EM algorithm for each *in silico* mixture. To assess the stability of EM-mediated estimation of methylated fraction at lower levels of genomic coverage (that is, the number of total sequenced bases in relation to the genome size), we downsampled the scores in the SM_SN_ distribution for the *in silico* mixtures of WGA and native molecules. The EM algorithm was then applied to these downsampled SM_SN_ score distributions.

The EM algorithm slightly, yet consistently, underestimates the native fraction in this mixture. To determine whether any non-normality in the methylated and non-methylated SM_SN_ score distributions might be causing the EM algorithm to underestimate the size of the modified fraction, we created Gaussian mixtures consisting of two entirely simulated normal distributions. To represent the non-methylated and methylated SM_SN_ scores, the distributions were simulated with α=2 and α=0, respectively (*σ*=0.5 for each). As an alternative, we also created mixtures using SM_SN_ values exclusively from WGA molecules where +2 was added to a subset of the SM_SN_ scores in order to get between 5 and 100% ‘methylated' SM_SN_ values, the distributions of which will retain any non-normality found in the WGA SM_SN_ distribution. The EM estimations of methylated fraction for these two types of simulated mixtures were both very similar and very accurate (Supplementary Fig. 2), indicating that non-normality of SM_SN_ score distributions is not the reason for the observed slight underestimation of native fraction. Instead, this evidence suggests that stably non-methylated 5′-CTGCAG sites in *E. coli* C227 are the reason for this phenomenon.

### Approximate SM_SN_ scores for *C. crescentus*

WGA sequencing was unavailable for analysis in this study, so the approximate SM_SN_ score shown in Fig. 3c is simply the mean of native molecule-, strand-, and position-specific log IPD values (rather than its difference from the mean of strand- and position-matched WGA log IPD values). This approximate SM_SN_ score is susceptible to IPD biases introduced by local sequence contexts, but provides a sufficient ability to resolve the methylated and non-methylated components in the bimodal distributions.

### FDR estimation for identifying methylated molecules

To assess the significance of observed methylated and non-methylated fractions based on SM_P_ scores, we established negative control SM_P_ distributions. When assessing the FDR for calling actively methylated molecules (for example, for the 5′-GWCAY motif in *H. pylori* J99), the negative control SM_P_ distribution is generated by running the SM_P_ method on WGA sequencing data. Alternatively, when assessing the FDR for calling non-methylated molecules (for example, for the 5′-TCAN_6_TRG/5′-CYAN_6_TGA and 5′-TCNNGA motifs in *H. pylori* J99), the negative control SM_P_ distribution is created by randomly shuffling IPD values among molecules to disperse the non-modified IPD values for the motif of interest. Given that the number of non-methylated IPD values is relatively low, this creates a control distribution of SM_P_ scores reflecting molecules that are mostly methylated at the motif of interest.

### Illumina MiSeq/HiSeq sequencing and analysis

For *C. salexigens* and the *H. pylori* J99 culture, Illumina-based MiSeq whole-genome libraries were prepared with an insert size of 600 bp as assessed by Agilent Bioanalysis using standard Illumina adapters and 8 PCR cycles. 2 × 300 bp paired-end sequencing was then conducted using version 3 commercial kits to assure the longest readlength possible. For the five single colonies of *H. pylori* J99 and the two mutant strains, both DNA and RNA were sequenced on HiSeq 2,500 with 100 bp paired-end reads. RNA of the wild-type *H. pylori* J99 strain was also sequenced using this method. Total RNAs were first treated with RiboZero bacterial Gram Negative ribosomal removal kit (Epicentre; MRZGN126) to obtain rRNA depleted RNAs.

The *E. coli* K12 MiSeq reads used for analysis of homopolymer indel rates (Fig. 4d) were downloaded from http://www.illumina.com/systems/miseq/scientific_data.ilmn.

The DNA sequencing reads from the *E. coli* K12, *C. salexigens*, and *H. pylori* J99 MiSeq runs were aligned to their respective references using *bwa mem*^68^ using default parameters. The resulting alignments were processed with the *samtools* package^69^ to obtain pileups for each genomic position. Read-level mismatch and insertion/deletion calls were parsed from the pileups by counting the number of occurrences of each type of variant at each genomic position.

To call differentially expressed genes from RNAseq data, we first mapped raw RNA reads for each sample to the Genbank reference (Accession number: AE001439). Reads that are mapped to rRNA and tRNAs were excluded. A gene was included for differential expression analysis if it had more than one count per million reads (CPM=1) in at least two samples. Differentially expressed genes are then called by the software program edgeR^70^ at *P*<0.001, corresponding to a FDR of 5%.

### Obtaining single colonies from *H. pylori* J99 stock strain

Roughly 20 μl *H. pylori* J99 strain frozen stock was spread on TSA plates with 5% sheep blood. After 96-h incubation at 37 °C in 5% CO_2_ conditions, five random single colonies were isolated and spread on new TSA plates for another 48-h incubation in the same conditions to enrich bacterial cultures for stock preparation and further tests. The five *H. pylori* strains obtained from the five random *H. pylori* J99 single colonies were named as HPXZ1383, HPXZ1384, HPXZ385, HPXZ1386 and HPXZ1387.

### Construction of *H. pylori* J99 MTase-knockout strains

Mutations of M.Hpy99XXII (*jhp1365*) and M.Hpy99XXI (*jhp1411*) were constructed by replacing the ORFs with *sacB*-*cat* cassettes (without disrupting the ORF of the upstream or downstream genes) through homologous recombination, as described below. A 0.9 Kb fragment upstream of *jhp1365* (jhp1365up) was obtained by PCR using primers JHP1365LF-SacII and JHP1365LR-SpeI (Supplementary Table 3), and a 0.6 Kb fragment downstream of *jhp1365* (jhp1365down) was obtained by PCR using primers JHP1365RF-SpeI and JHP1365RR-PstI (Supplementary Table 3). Fragments jhp1365up and jhp1365down were digested with *Sac*II/*Spe*I and *Spe*I/*Pst*I, respectively, and then ligated together into *Sac*II/*Pst*I-digested pGEM-T Easy, creating pXZ577 (Supplementary Table 4). Similarly, a 0.9 Kb fragment upstream of *jhp1411* (jhp1411up) was obtained by PCR using primers JHP1411LF-SacII and JHP1411LR-SpeI (Supplementary Table 3), and a 0.7 Kb fragment downstream of *jhp1411* (jhp1411down) was obtained by PCR using primers JHP1411RF-SpeI and JHP1411RR-PstI (Supplementary Table 3). Fragments jhp1411up and jhp1411down were digested with *Sac*II/*Spe*I and *Spe*I/*Pst*I, respectively, and then ligated together into *Sac*II/*Pst*I-digested pGEM-T Easy, creating pXZ579 (Supplementary Table 4). The *sacB-cat* cassette, conferring chloramphenicol resistance (Cm^R^), was obtained by PCR using pXZ032 (ref. 64) as template and primers SC-F-XbaI and SC-R-XbaI (Supplementary Table 3). The *sacB*-*cat* cassette was digested with *Xba*I, and then ligated with *Spe*I-digested pXZ577 and pXZ579, creating pXZ578 and pXZ580, respectively (Supplementary Table 4). All plasmid constructions were confirmed by sequencing using primers PGEMTe-seqF and PGEMTe-seqR (Supplementary Table 3).

The wild type *H. pylori* strain J99 was transformed to Cm^R^ with these plasmids, pXZ578 and pXZ580, to create mutant HPXZ1398 (*jhp1365*::*sacB-cat*) and HPXZ1401 (*jhp1411*::*sacB-cat*), respectively (Supplementary Table 4), as previously described^64^. Genomic DNA of these mutants was isolated and PCR was performed to confirm that HPXZ1398 and HPXZ1401 carry the correct mutation of *jhp1365*::*sacB-cat* and j*hp1411*::*sacB-cat*, respectively, with *sacB-cat* cassette-specific primer Catup and genome locus-specific primer JHP1365RR-PstI or JHP1411RR-PstI (Supplementary Table 3).

### Sensitivity and specificity of SM_P_ methylation detection

To measure the sensitivity and specificity of the SM_P_ scores for assessing methylated molecules (and by extension MTase activity), we applied the method to the 5′-GATC motif in *H. pylori* J99. The responsible MTase, M.Hpy99VI, is not suspected to be phase-variable and SM_SN_ scores revealed a very small non-methylated fraction (Fig. 3a). We therefore expect that nearly 100% of molecules in the sample will be targeted for methylation at the 5′-GATC motif. As the sensitivity and specificity curves show (Supplementary Fig. 18), the power of the SM_P_ scores to detect methylated molecules increases with an increasing threshold on the number of motif sites present on the molecule. Although lower values of this threshold have limited ability to correctly identify methylated molecules, some methylated motifs are distributed too sparsely in the genomes to be interrogated using higher thresholds. Shorter motifs are usually present at higher densities in a genome, but there are some exceptions to this rule (Supplementary Table 2). Therefore, we suggest using the number of motif sites on each molecule (rather than motif length) as the threshold for SM_P_ analysis.

## Additional information

**Accession codes:** Sequence data have been deposited at the NCBI Sequence Read Archive (SRA) with BioProject under project ID PRJNA281410 and the DNA Database of Japan under accession code SRP057274. The assembled reference genomes used in this study have been deposited in GenBank under the accession codes CP011331 (E. coli C227) and CP011330 (H. pylori J99).

**How to cite this article:** Beaulaurier, J. *et al.* Single molecule-level detection and long read-based phasing of epigenetic variations in bacterial methylomes. *Nat. Commun.* 6:7438 doi: 10.1038/ncomms8438 (2015).

## Supplementary Material

Supplementary Information Supplementary Figures 1-18 and Supplementary Tables 1-6
