# Supplementary material for: Single molecule-level detection and long read-based phasing of epigenetic variations in bacterial methylomes
Source: Nat Commun. 2015 Jun 15;6:7438. doi: 10.1038/ncomms8438 (PMC4490391; doi:10.1038/ncomms8438)
Supplement: Supplementary Information — Supplementary Figures 1-18 and Supplementary Tables 1-6 [file ncomms8438-s1.pdf]

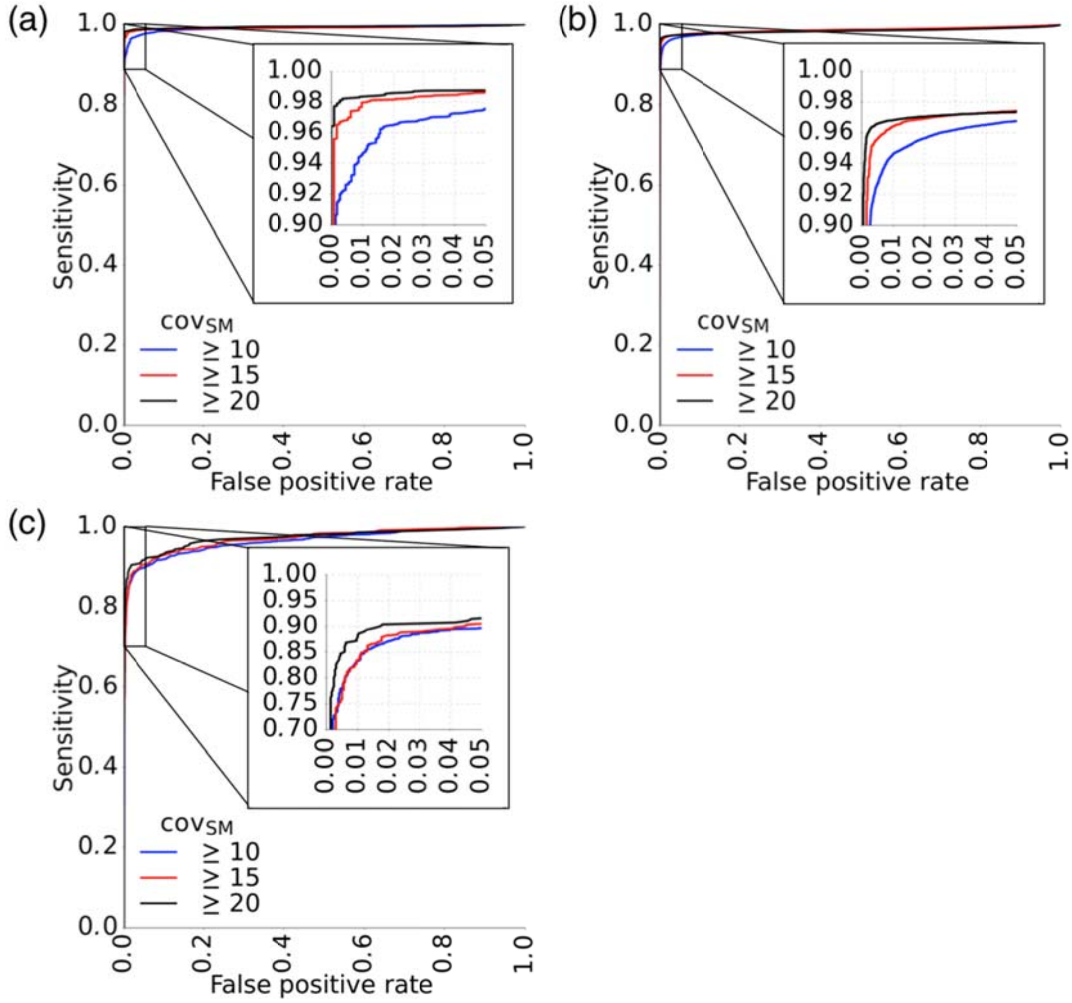

Supplementary Figure 1: Sensitivity and specificity of the SM_{SN} method for detecting DNA modifications using three thresholds for minimum single-molecule coverage (cov_{SM}): **(a)** 6mA modifications in the 5'-ACCACC motif in *E. coli* C227, **(b)** 6mA modifications in the 5'-GATC motif in *E. coli* C227, and **(c)** 4mC modifications in the 5'-CGWCG motif in *H. pylori* J99.

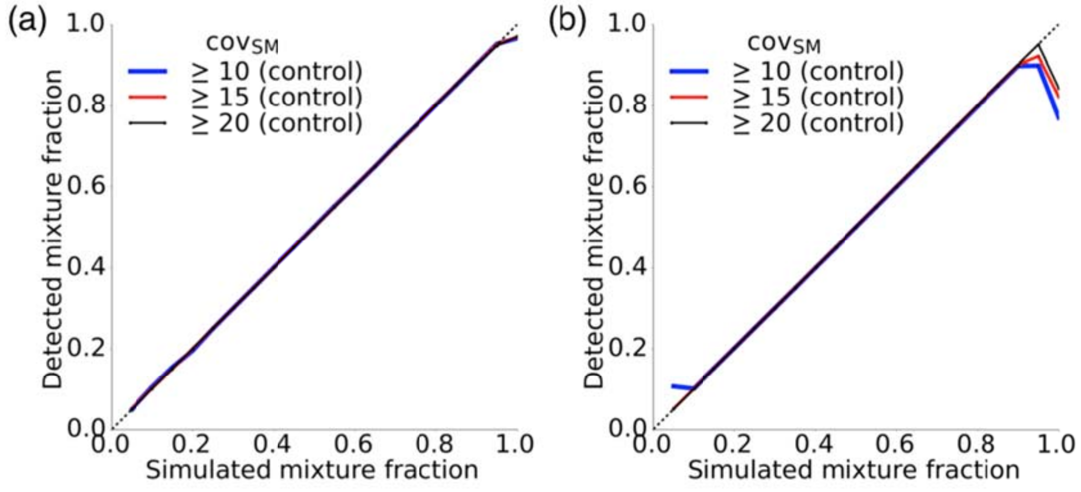

Supplementary Figure 2: Two alternative approaches for generating simulated modified and non-modified distributions used to test the ability of the SM_{SN} scores to estimate the size of the modified fraction. Instead of mixing varying proportions of WGA and native molecules, we instead **(a)** simulated two normal distributions centered around the mean SM_{SN} scores for modified ($SM_{SN} = 2$) and non-modified ($SM_{SN} = 0$) adenine residues and varied their relative proportions, and **(b)** exclusively used WGA molecules, but simulated the presence of a modification by adding 2 to the WGA SM_{SN} scores in a varying number of WGA molecules.

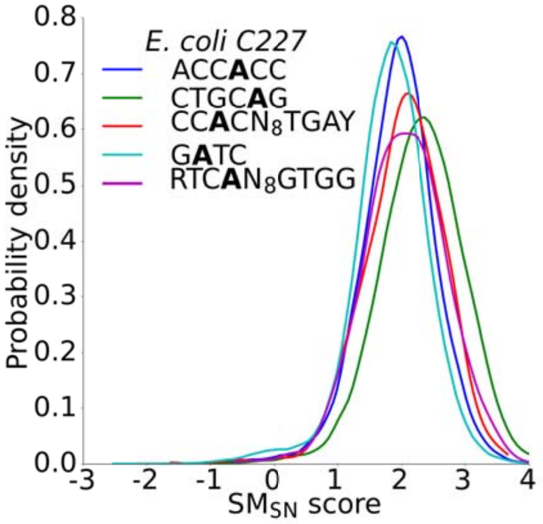

Supplementary Figure 3: SM_{SN} score distributions ($cov_{SM} \geq 10$) for all five 6mA motifs in *E. coli* C227.

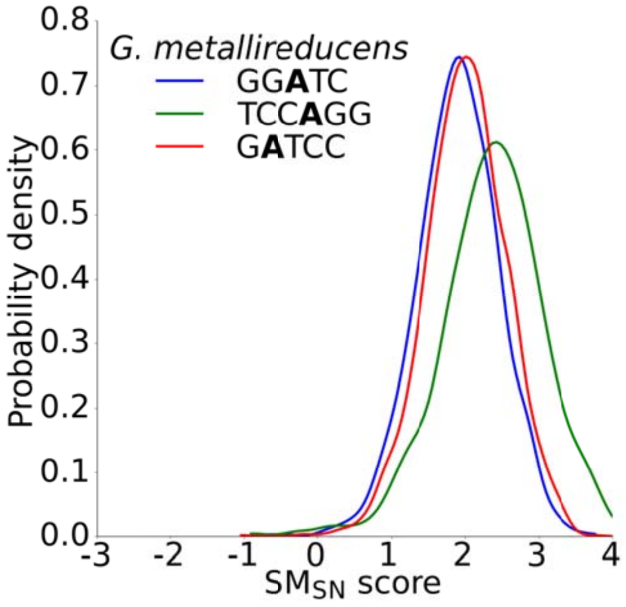

Supplementary Figure 4: SM_{SN} score distributions ($cov_{SM} \geq 10$) for all three 6mA motifs in *G. metallireducens*.

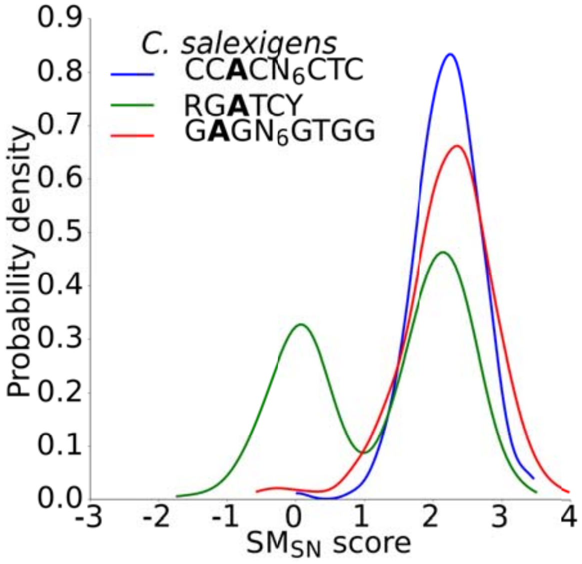

Supplementary Figure 5: SM_{SN} score distributions ($cov_{SM} \geq 10$) for all three 6mA motifs in *C. salexigens*.

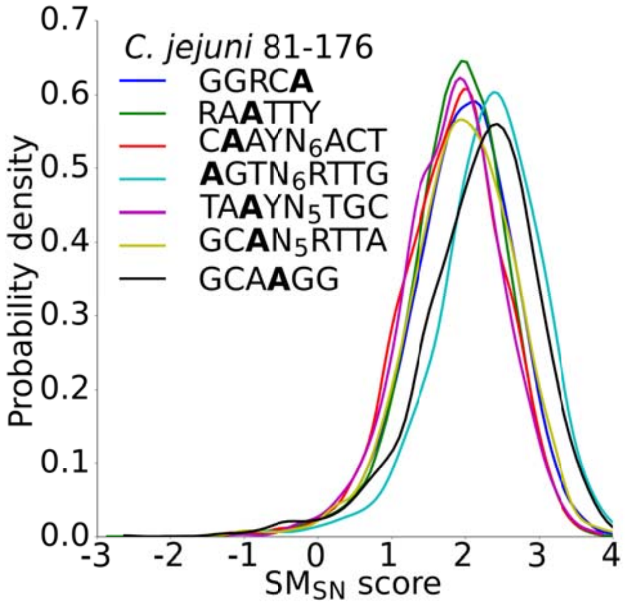

Supplementary Figure 6: SM_{SN} score distributions ($cov_{SM} \geq 5$) for all seven 6mA motifs in *C. jejuni* 81-176.

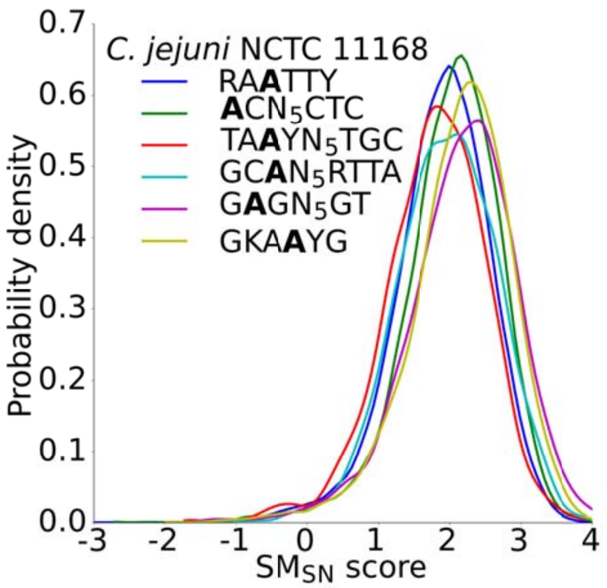

Supplementary Figure 7: SM_{SN} score distributions ($cov_{SM} \geq 5$) for all six 6mA motifs in *C. jejuni* NCTC 11168.

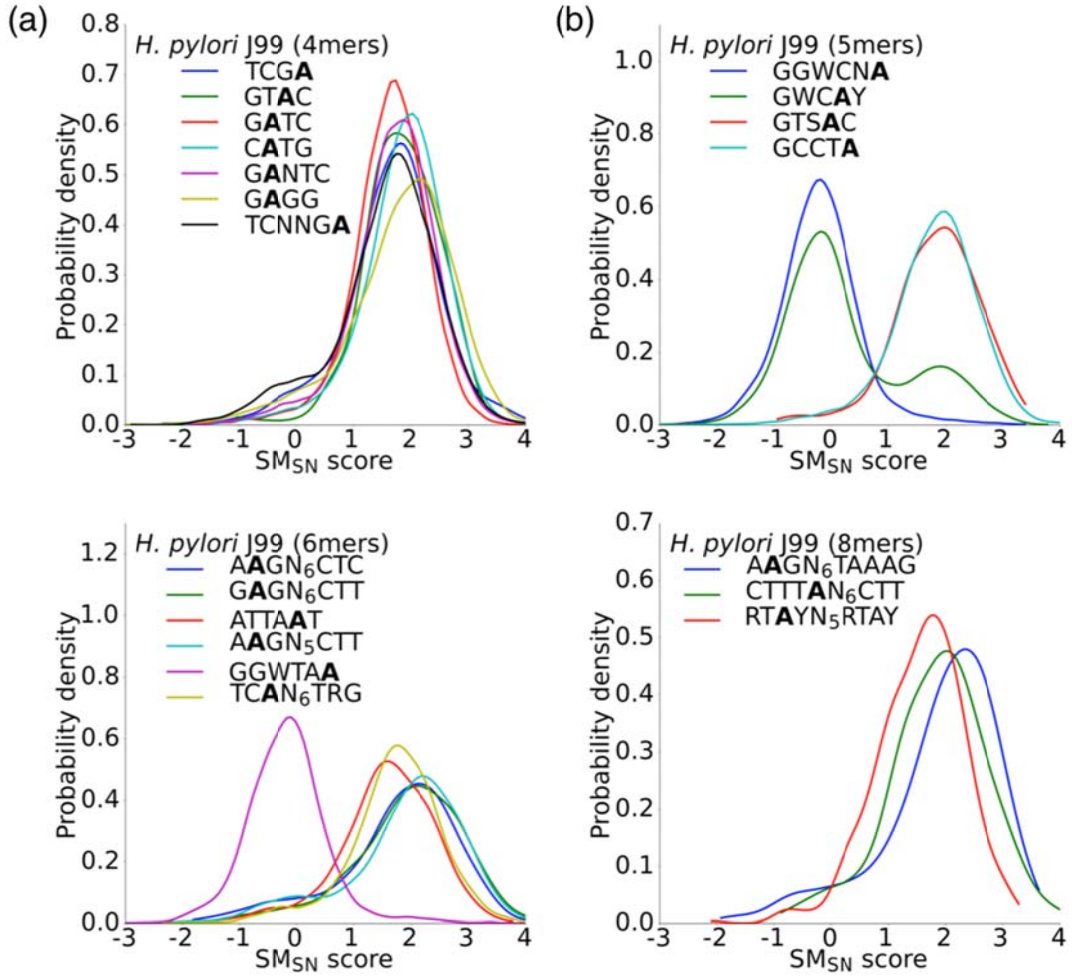

Supplementary Figure 8: SM_{SN} score distributions ($cov_{SM} \geq 5$) in *H. pylori* J99 for **(a)** all 4mer 6mA motifs, **(b)** all 5mer 6mA motifs, **(c)** all 6mer 6mA motifs, and **(d)** all 8mer 6mA motifs.

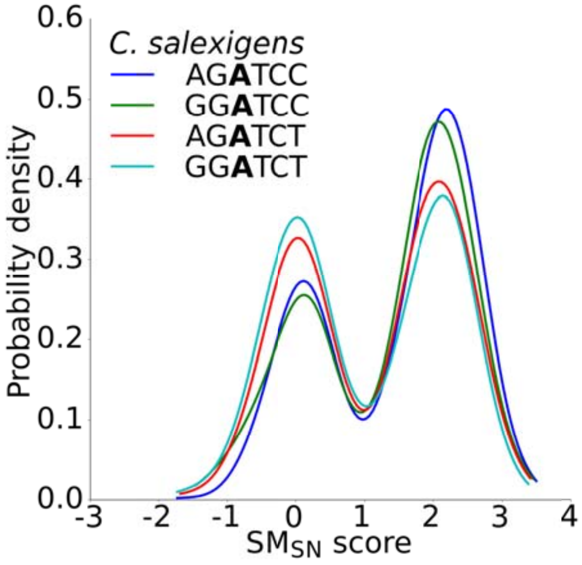

Supplementary Figure 9: The SM_{SN} score distributions ($cov_{SM} \geq 10$) for each specification of the degenerate 5'-RGATCY 6mA motif show similar levels of global heterogeneity.

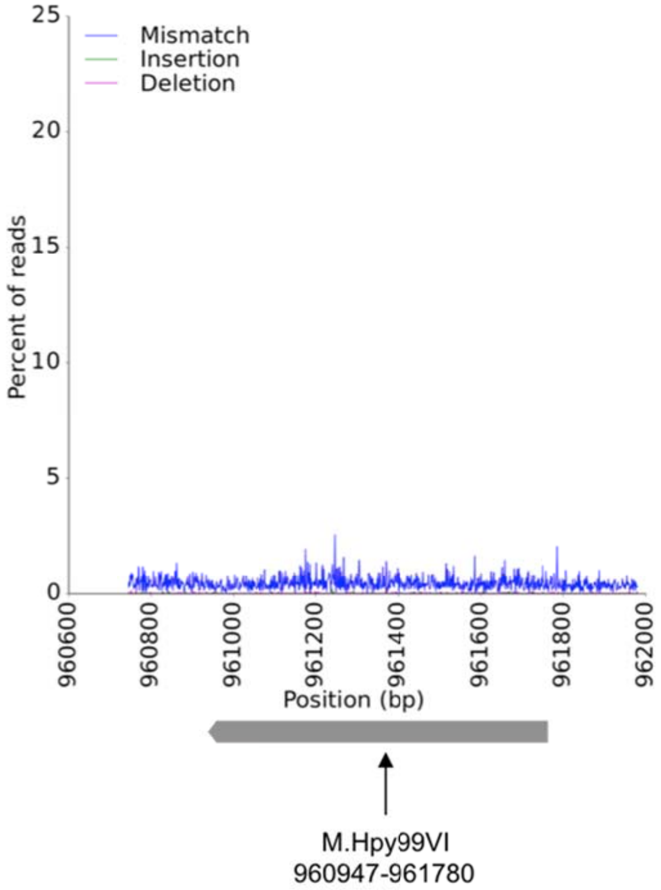

Supplementary Figure 10: Read-level MiSeq mismatch and insertion/deletion calls in the coding region (+/-200bp) of the 5'-GATC-targeting *M.Hpy99VI* gene in *H. pylori* J99.

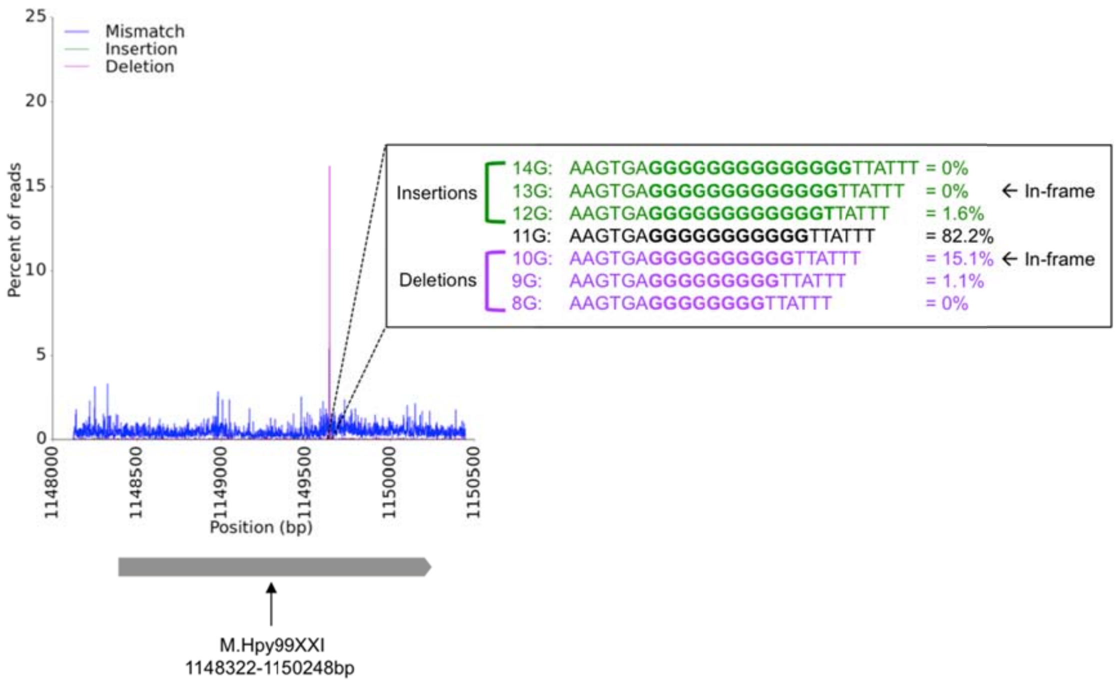

Supplementary Figure 11: Read-level MiSeq mismatch and insertion/deletion calls in the coding region (+/-200bp) of the 5'-GWC<u>A</u>Y-targeting *M.Hpy99XXI* gene in *H. pylori* J99.

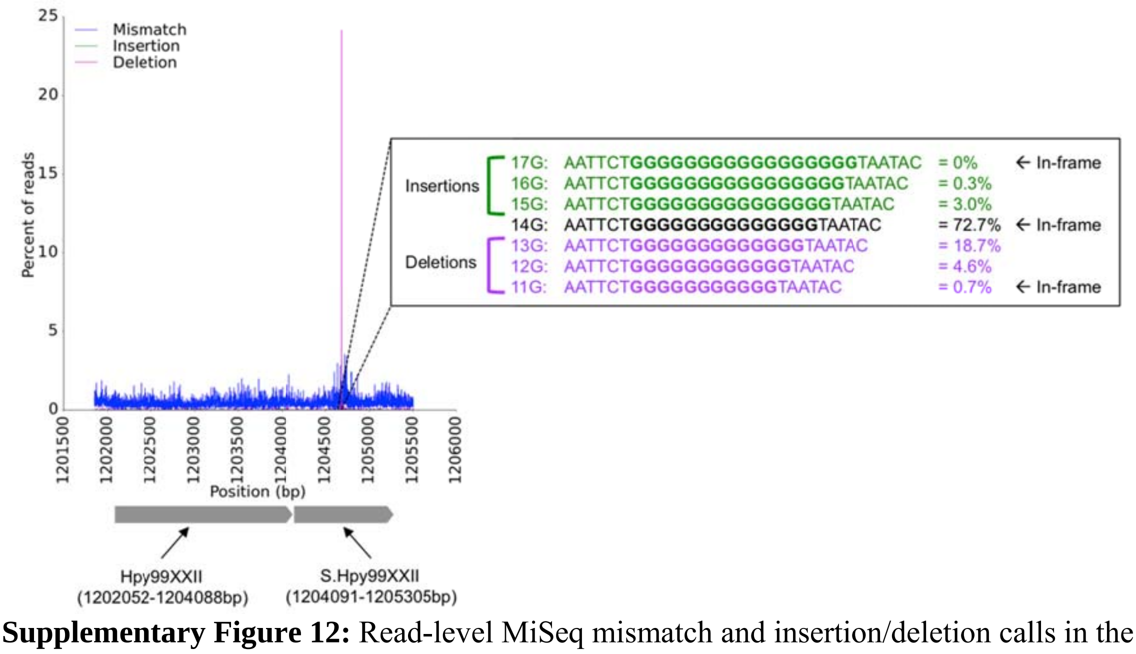

Supplementary Figure 12: Read-level MiSeq mismatch and insertion/deletion calls in the coding region (+/-200bp) of the 5'-TC<u>A</u>N_6TRG/5'-CY<u>A</u>N_6TGA-targeting *Hpy99XXII* and *S.Hpy99XXII* genes in *H. pylori* J99.

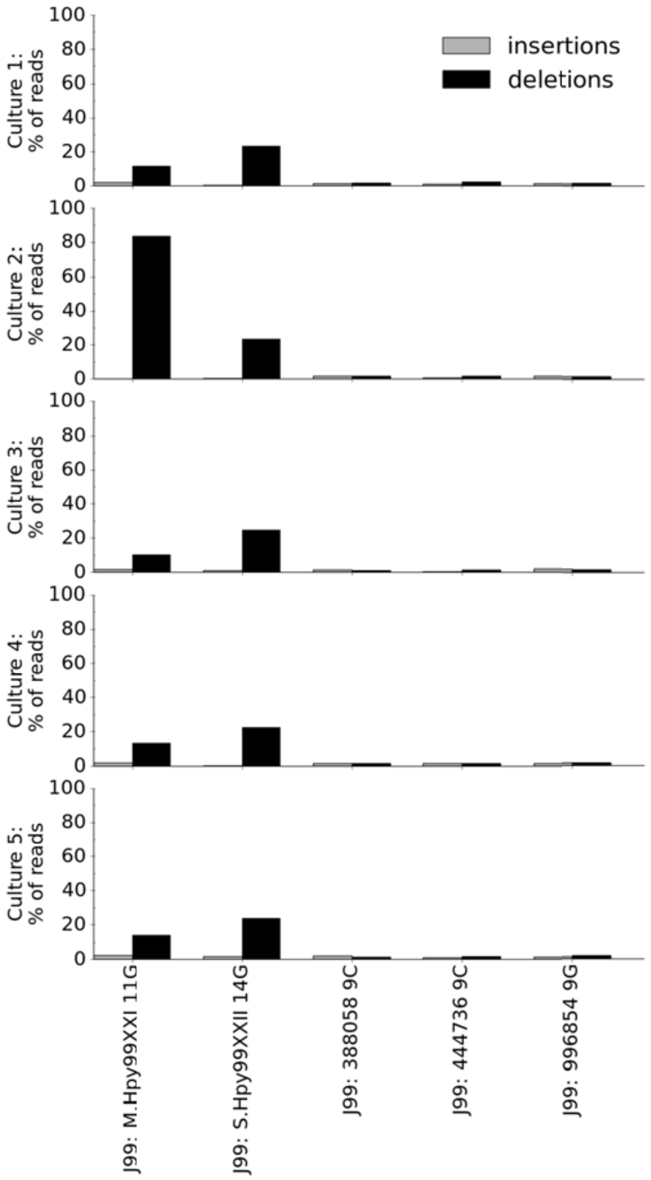

Supplementary Figure 13: Percentage of reads with insertion/deletion calls at multiple C/G homopolymer loci in five clonal cultures isolated from the original *H. pylori* J99 lab strain. The significant percentage of deletions in two specific poly-G regions within the coding sequences of *M.Hpy99XXI* and *S.Hpy99XXII* stand apart from the minimal length variation observed at three other 9C/G homopolymer loci in *H. pylori* J99. This suggests that the homopolymer length variation in *M.Hpy99XXI* and *S.Hpy99XXII* occurs even in clonal samples rapidly within 144 hours and is not the result of diverging, stable subpopulations within the original cell culture. One of the five colonies has a large percentage of deletions in *M.Hpy99XXI* (i.e. mostly 10G homopolymer rather than 11G), which suggests that this single colony is likely derived from a parent cell with a 10G homopolymer

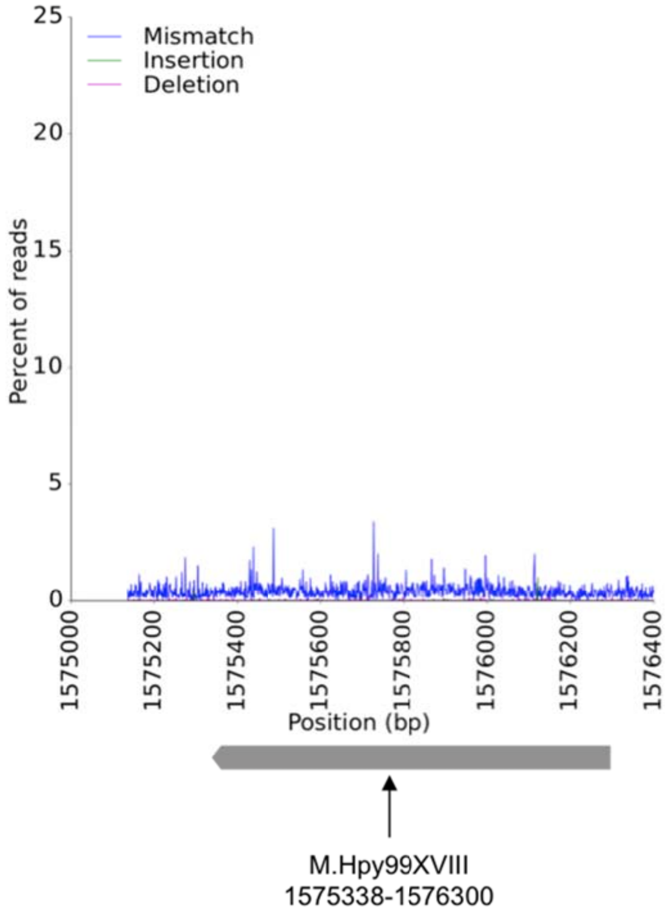

Supplementary Figure 14: Read-level MiSeq mismatch and insertion/deletion calls in the coding region (+/-200bp) of the 5'-TCNNGA-targeting *M.Hpy99XVIII* gene in *H. pylori* J99.

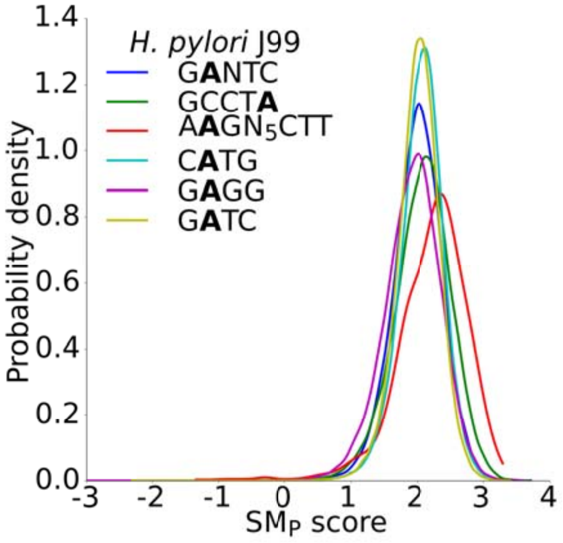

Supplementary Figure 15: SMP score distributions (at least 10 motif sites per molecule) for all 6mA motifs in *H. pylori* J99 (excluding 5'-GWCAY, 5'-TCAN_6TRG, and 5'-TCNNGA) that contained at least ten distinct motif sites on at least 500 molecules.

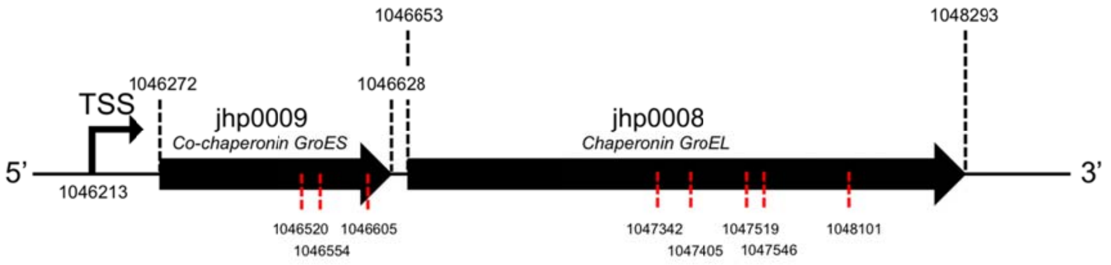

Supplementary Figure 16: Distribution of 5'-GWCAY motif sites in the two-gene operon encoding GroES and GroEL that are significantly up-regulated in HPXZ1401, from the left to the right (5'➔3').

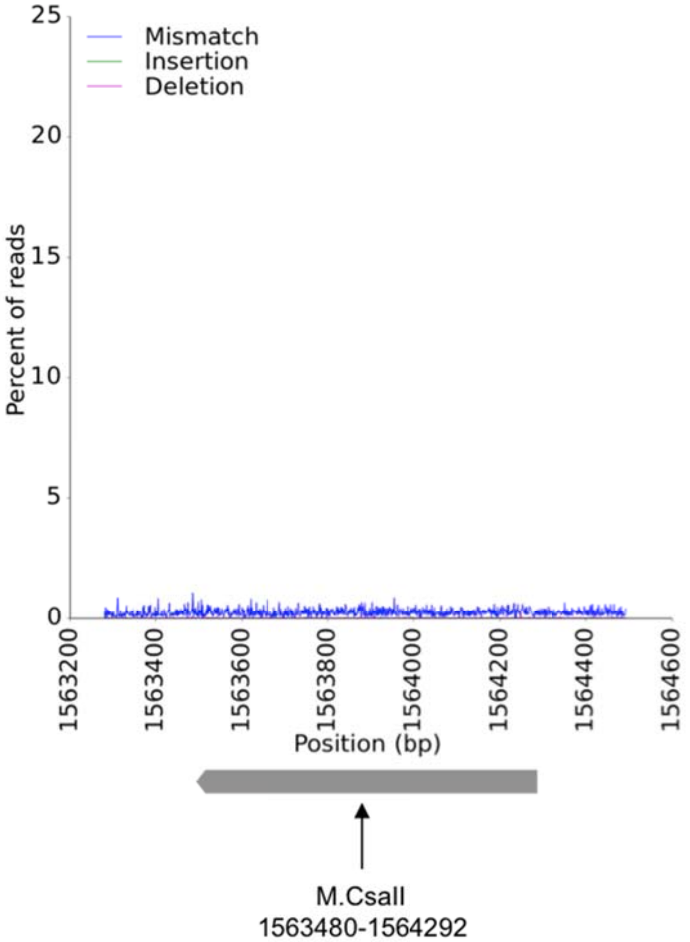

Supplementary Figure 17: Read-level MiSeq mismatch and insertion/deletion calls in the coding region (+/-200bp) of the 5'-RG<u>A</u>TCY-targeting *M.CsaI* gene in *C. salexigens*.

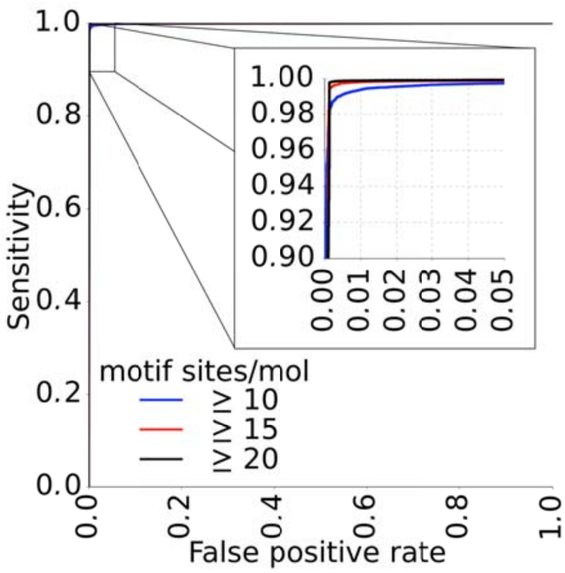

Supplementary Figure 18: Sensitivity and specificity of the SM_P method for detecting molecules that are methylated at the 5’-GATC motif in *H. pylori* J99.

Supplementary Table 1: SMRT sequencing statistics.

**Organism**	**Sample type**	**Library**	**Reference GenBank accession**	**Mean insert length**	**Coverage**	**Mean read length**	**Mean subread length**
Escherichia coli O104:H4 C227							
	Native	Short	CP011331	202	402.46	5609	172
	WGA	Short	CP011331	197	97.33	6091	167
Chromohalobacter salexigens							
	Native	Short	NC_007963.1	183	78.57	8119	183
		Long	NC_007963.1	3357	49.13	5233	3357
	WGA	Short	NC_007963.1	180	184.76	9023	180
Helicobacter pylori J99							
	Native	Short	NC_000921.1	171	71.31	4735	168
		Long	CP011330	7304	1093.73	7965	6871
	WGA	Short	NC_000921.1	195	215.19	6873	167
		Long	CP011330	3978	390.35	4957	3978

Supplementary Table 2: Genome-wide motif counts.

**Organism (reference GenBank accession)**	**Motif**	**Count**
Escherichia coli O104:H4 C227 (CP011331)		
	ACCACC	4321
	CTGCAG	2528
	CCACNNNNNNNNTGAY	478
	GATC	42186
	RTCANNNNNNNNGTGG	478
Chromohalobacter salexigens (NC_007963.1)		
	CCACNNNNNNCTC	784
	RGATCY	5084
	GAGNNNNNNGTGG	784
Geobacter metallireducens (NC_007517.1)		
	GGATC	11347
	TCCAGG	5821
	GATCC	11347
Campylobacter jejuni 81-176 (NC_008787.1)		
	GGRCA	1849
	RAATTY	26876
	CAAYNNNNNNACT	309
	AGTNNNNNNRTTG	309
	TAAYNNNNNTGC	460

	GCANNNNNRTTA	460
	GCAAGG	699
Campylobacter jejuni NCTC 11168 (NC_018521.1)		
	RAATTY	27280
	ACNNNNNCTC	1062
	TAAYNNNNNTGC	588
	GCANNNNNRTTA	469
	GAGNNNNNGT	1062
	GKAAYG	1255
Helicobacter pylori J99 (NC_000921.1)		
	TCGA	674
	GTAC	368
	GATC	10958
	CATG	15120
	GANTC	5516
	GAGG	5001
	TCNNGA	3906
	GGWCNA	1635
	GWCAY	5101
	GTSAC	210
	GCCTA	3106
	AAGNNNNNNCTC	611
	GAGNNNNNNCTT	611
	ATTAAT	854
	AAGNNNNNCTT	1582
	GGWTAA	2676
	AAANNNNNNTGG	2211
	CCANNNNNNTTT	2211
	TCANNNNNNTRG	1978
	CYANNNNNNTGA	1978
	AAGNNNNNNTAAAG	286
	CTTTANNNNNNCTT	286
	RTAYNNNNNRTAY	362
Helicobacter pylori J99 (CP011330)		
	TCGA	690
	GTAC	368
	GATC	11140
	CATG	15336
	GANTC	5588
	GAGG	5120
	TCNNGA	4056
	GGWCNA	1688
	GWCAY	5263
	GTSAC	208

	GCCTA	3214
	AAGNNNNNNCTC	639
	GAGNNNNNNCTT	639
	ATTAAT	862
	AAGNNNNNCTT	1626
	GGWTAA	2765
	AAANNNNNNTGG	2256
	CCANNNNNNTTT	2256
	TCANNNNNNTRG	2041
	CYANNNNNNTGA	2041
	AAGNNNNNNTAAAG	303
	CTTTANNNNNNCTT	303
	RTAYNNNNNRTAY	372

Supplementary Table 3: Oligonucleotides used in this study.

Oligonucleotide	Primer sequence (5'=>3')*
SC-F-XbaI	GCTCTAGATATAAGCCCATTTTCATGCTCC
SC-R-XbaI	GCTCTAGACTCGAGGCGTGATATAGATTGAAAAGTG
JHP1365LF-SacII	TCCCCGCGGCGCATGAATTGTCCCGCATACCATG
JHP1365LR-SpeI	GGACTAGTCCAAACGCCCCCAAAGTGATTGGCC
JHP1365RF-SpeI	GGACTAGTGGCTCAATAGATTGAACTTTATTC
JHP1365RR-PstI	AAACTGCAGGAATGCGTTGTTTAACCTGTTTGAAG
JHP1411LF-SacII	TCCCCGCGGGTAATTGATCGTGGCTTCATTTTG
JHP1411LR-SpeI	GGACTAGTGGGGGTAGAGGTGAAAATCAAATTC
JHP1411RF-SpeI	GGACTAGTCTTTATTTTGCATCTCTATCCTTT
JHP1411RR-PstI	AAACTGCAGGATGGAGAAAATTAGCGAAGAAATCG
Catup	TGGATGAATTACAAGACT
PGEMTe-seqF	GCTGGCGAAAGGGGGATGTGCTGCAAG
PGEMTe-seqR	CGCTCGCCGCAGCCGAACGA
	* Restriction sites underlined; *Xba*I (TCTAGA), *Sac*II (CCGCGG), *Spe*I (ACTAGT), *Pst*I (CTGCAG).

Supplementary Table 4: Plasmids and bacterial strains used.

Plasmid or strain	Relevant characteristics*	Source or reference
pGEM-T easy	Vector to construct gene mutations, Ap^R	Promega
pXZ032	pGEM-T easy, carrying *sacB-cat* cassette, Ap^R, Cm^R	REF 64
pXZ577	pGEM-T easy, *jhp1365* upstream flanking region directly fused with *jhp1365* downstream flanking region, Ap^R	This work
pXZ578	pXZ577, *sacB-cat* cassette inserted between *jhp1365* upstream and downstream flanking region to construct Δ *jhp1365*::*sacB-cat* mutation, Ap^R, Cm^R	This work
pXZ579	pGEM-T easy, *jhp1411* upstream flanking region directly fused with *jhp1411* downstream flanking region, Ap^R	This work
pXZ580	pXZ579, *sacB-cat* cassette inserted between *jhp1411* upstream and downstream flanking region to construct Δ *jhp1411*::*sacB-cat* mutation, Ap^R, Cm^R	This work
E. coli		

XL1-blue	Host for cloning plasmid	Stratagene
H. pylori		
J99	USA-origin isolate	REF 53
HPXZ1398	J99 Δ*jhp1365*::*sacB-cat*	This work
HPXZ1401	J99 Δ*jhp1411*::*sacB-cat*	This work
	*Ap^R and Cm^R represent ampicillin-resistant and chloramphenicol-resistant, respectively.	

Supplementary Table 5: Differentially expressed genes (FDR < 0.05) in HPXZ1401 vs. HPXZ1383-1387. Start/End positions listed for the *H. pylori* J99 HGAP reference assembly.

**Induced gene list**											
**ORF**	**Gene**	**Start**	**End**	**Strand**	**Description**	**log2 fold change (KO vs. WT)**	**p value**	**FDR**	**Distance to 5 nearest upstream GWCAY sites**	**GWCAY positions in coding region**	**Distance to 5 nearest downstream GWCAY sites**
Flagellum related											
jhp0688	*flaG*	768725	769084	+	flagellar protein FlaG	3.44	7.58E-04	4.10E-02	-760,-1222,-1300,-2697,-3106,-3673		+856,+1282,+1531,+1549,+1840,+2088
jhp0804	*flgE*	881778	883934	-	flagellar hook protein FlgE	4.12	1.50E-10	2.19E-07	-283,-381,-652,-2028,-2958,-2973	883237,883141,882916,882643	+252,+357,+462,+684,+1326,+1544
jhp1467	*flgB*	1617081	1617503	-	flagellar basal-body rod protein FlgB	2.81	1.51E-05	2.01E-03	-205,-623,-1704,-1767,-2154,-2919	1617193	+819,+1295,+2588,+3637,+4282,+4331
chaperone											
jhp0008	*groEL*	7758	9398	-	molecular chaperone GroEL	2.32	4.20E-04	2.83E-02	-43,-94,-128,-955,-2114,-3797	8704,8641,8527,8500,7945	+461,+665,+738,+1393,+3215,+3302
jhp0009	*groES*	9423	9779	-	co-chaperonin GroES	3.58	7.99E-09	2.34E-06	-574,-1733,-3416,-3911,-4080,-5505	9526,9492,9441	+719,+782,+896,+923,+1478,+2126
jhp0102	*grpE*	114404	114979	-	heat shock protein GrpE	3.20	9.98E-04	4.55E-02	-175,-430,-1244,-1457,-1715,-1779		+2312,+3723,+4146,+4347,+5116,+5655
jhp0103	*hrcA*	114979	115779	-	heat-inducible transcription repressor	3.11	1.04E-06	1.69E-04	-444,-657,-915,-979,-1786,-2410	115409,115154	+2887,+4298,+4721,+4922,+5691,+6230
DNA processing											
jhp1266	*ung*	1383952	1384653	-	uracil-DNA glycosylase	2.61	1.08E-03	4.55E-02	-103,-472,-517,-1058,-1352,-1745		+327,+441,+648,+1056,+1226,+1934
Metobolism, transporter, and other											
jhp0099	*cysK*	110644	111561	-	cysteine synthase	2.37	1.08E-03	4.55E-02	-531,-3593,-3848,-4662,-4875,-5133	110681	+386,+587,+1356,+1895,+2114,+2414
jhp0126		140867	142312	-	predicted iron-sulfur protein	2.35	9.36E-04	4.55E-02	-155,-168,-637,-998,-1744,-2677	142215,140916	+567,+618,+1908,+3093,+3509,+4000
jhp0545	*bioF*	593501	594622	-	8-amino-7-oxononanoate synthase	3.82	4.25E-09	1.55E-06	-162,-324,-744,-828,-3459,-3473	594315,593901,593838,593565	+262,+304,+1372,+2089,+2227,+5699
jhp0560	*aspS*	621872	623611	+	aspartyl-tRNA synthetase	2.23	9.97E-04	4.55E-02	-248,-965,-3689,-4280,-6431,-10466	621881,622168,623242	+1163,+1672,+2082,+2829,+4577,+4841
jhp0627	*feoB*	697655	699583	-	ferrous iron transport protein B	2.55	1.24E-03	4.73E-02	-661,-1320,-1374,-1574,-2572,-3274	699264	+147,+294,+1202,+1250,+2150,+2201
jhp0640	*dgkA*	714369	714755	+	diacylglycerol kinase	2.25	5.07E-04	3.16E-02	-1047,-1517,-1721,-1775,-2643,-4883	714491,714671	+13,+273,+450,+480,+2136,+2241
jhp0653		731774	732496	-	ABC transporter ATP-binding protein	2.88	1.11E-04	1.19E-02	-269,-562,-2204,-3273,-3813,-3970	731973	+4,+658,+2289,+3086,+4094,+4266
jhp0935		1037343	1037885	-	Predicted partitioning protein A	2.48	5.20E-04	3.16E-02	-358,-1548,-1560,-1745,-2081,-2402	1037671,1037593,1037437	+287,+456,+858,+960,+2009,+2513
jhp1093	*pgi*	1211430	1213067	+	glucose-6-phosphate isomerase	2.44	1.69E-04	1.64E-02	-550,-4094,-4303,-4746,-4864,-5609	1212530,1212911	+189,+683,+1233,+1284,+2965,+3649
jhp1324	*kpsF*	1453242	1454231	+	Predicted polysialic acid capsule expression protein	3.34	6.54E-08	1.59E-05	-1,-299,-671,-860,-1580,-6136		+116,+2023,+2377,+2780,+4027,+5490

Unknown function										
jhp0143		158730	159005	+	hypothetical protein	3.45	3.50E-04	2.64E-02	-1477,-1540,-2436,-2880,-3099,-5118		+439,+1142,+1652,+1757,+2140,+2225
jhp0153		168125	168598	-	hypothetical protein	4.10	1.09E-03	4.55E-02	-455,-844,-1253,-1717,-1753,-2285		+301,+1212,+2564,+3560,+4873,+5098
jhp0619		687381	688148	-	hypothetical protein, predicted integral membrane protein	3.21	7.49E-05	9.12E-03	-495,-606,-669,-1735,-1963,-3289	687769	+80,+383,+1085,+1671,+3068,+3616
jhp0654		732509	732910	-	hypothetical protein	3.78	1.14E-04	1.19E-02	-148,-1790,-2859,-3399,-3556,-3568	732765	+536,+739,+1393,+3024,+3821,+4829
jhp0986		1096410	1097078	+	hypothetical protein	4.15	1.77E-09	1.29E-06	-125,-317,-368,-662,-746,-1525		+1043,+1283,+2074,+3631,+4488,+6066
jhp0987		1097080	1097838	+	hypothetical protein	4.01	3.77E-09	1.55E-06	-795,-987,-1038,-1332,-1416,-2195		+283,+523,+1314,+2871,+3728,+5306
jhp1163		1295719	1295949	+	hypothetical protein	2.71	1.49E-05	2.01E-03	-201,-345,-1278,-3697,-3816,-4813	1295814	+9,+1955,+2801,+3108,+3817,+8756
jhp1319		1448519	1449139	+	hypothetical protein	3.68	6.59E-07	1.20E-04	-1413,-2232,-2296,-2434,-4834,-5522		+2523,+3243,+3432,+3804,+4102,+5208
**Repressed gene list**										
**ORF**	**Gene**	**Start**	**End**	**Strand**	**Description**	**log2 fold change (KO vs. WT)**	**p value**	**FDR**	**Distance to 5 nearest upstream GWCAY sites**	**GWCAY positions in coding region**	**Distance to 5 nearest downstream GWCAY sites**
DNA processing											
jhp0811	*ruvC*	891084	891557	-	Holliday junction resolvase	-3.29	6.46E-04	3.63E-02	-1343,-1516,-1810,-2115,-3739,-4861	891443	+98,+1016,+1534,+1629,+2914,+3184
jhp0826	*tnpB*	908966	910294	-	IS606 transposase	-3.62	1.20E-03	4.73E-02	-575,-1442,-1774,-3148,-3295,-3688	909750,909627,909585,909401,909060	+403,+1497,+1918,+3654,+3735,+4531
jhp0931	*topA_3*	1032846	1034906	+	topoisomerase I	-4.33	4.25E-04	2.83E-02	-503,-935,-2639,-3263,-3578,-3905	1032860	+590,+675,+758,+1445,+1583,+1607
Metobolism											
jhp0006	*panC*	3235	4065	+	pantoate--beta-alanine ligase	-5.29	1.80E-04	1.65E-02	-438,-444	3735	+89,+306,+631,+1733,+2587,+2717
jhp0709	*amiA*	786502	787911	-	N-acetylmuramoyl-L-alanine amidase	-4.14	1.26E-03	4.73E-02	-410,-3136,-3394,-4384,-4735,-5095	787533,787097,787016	+1020,+1141,+1149,+1276,+1617,+2204
jhp0968	*cfa*	1072958	1074127	+	cyclopocyclopropane fatty acid synthase	-3.45	1.17E-03	4.73E-02	-211,-3983,-4061,-4442,-7279,-7375	1073107,1073119,1073377,1073926	+1038,+1359,+2211,+3337,+3515,+4177
jhp1023		1134753	1135505	+	short chain alcohol dehydrogenase	-3.78	8.19E-04	4.28E-02	-102,-249,-906,-1281,-3269,-4847	1135058,1135067,1135388	+745,+785,+1071,+3399,+4852,+4868
Unknown function											
jhp0869		961971	962456	+	hypothetical protein	-3.49	5.73E-04	3.35E-02	-2593,-2908,-5249,-5567,-5588,-7961		+18,+352,+402,+2685,+5238,+5282
jhp0927		1023777	1025174	+	hypothetical protein	-4.97	3.61E-04	2.64E-02	-2612,-2774,-4635,-7078,-8448,-8651	1024973	+451,+1361,+1937,+2258,+2357,+2489
jhp0933		1035401	1036204	+	hypothetical protein	-5.22	2.91E-04	2.36E-02	-2541,-3058,-3490,-5194,-5818,-6133	1035496,1035581,1035664	+147,+285,+309,+1926,+2043,+2214
jhp0934		1036277	1037296	-	hypothetical protein	-5.23	2.37E-04	2.04E-02	-141,-297,-375,-947,-2137,-2149	1037056,1036887,1036485,1036383	+943,+1447,+2074,+2272,+3283,+5291
jhp1397		1528923	1529639	-	hypothetical protein	-4.19	8.57E-04	4.32E-02	-379,-552,-1642,-1657,-2224,-2372	1529518,1529158	+1394,+1892,+3425,+3611,+3638,+3821

Supplementary Table 6: Differentially expressed genes (FDR<0.05) in HPXZ1398 vs. HPXZ1383-1387. Start/End positions listed for the *H. pylori* J99 HGAP reference assembly.

**Induced gene list**											
**ORF**	**Gene**	**Start**	**End**	**Strand**	**Description**	**log2 fold change (KO vs.**	**p value**	**FDR**	**Distance to 5 nearest upstream**	**TCANNNNNNTRG positions in coding region**	**Distance to 5 nearest downstream**

						WT)			TCANNNNN NTRG sites		TCANNNNNN TRG sites
Flagellum related											
jhp0804	*flgE*	881778	883934	-	flagellar hook protein FlgE	3.58	1.60E-08	3.58E-06	-716,-4949,-5395,-5582,-7217,-7442	883641	+5645,+6839,+8596,+12802,+15209,+16300
DNA processing											
jhp0785	*hsdS_2*	864828	865451	-	type I restriction enzyme S genes	2.29	7.13E-04	3.24E-02	-27,-1118,-3525,-7731,-9488,-10682		+219,+2264,+2678,+4138,+4882,+5035
jhp1438	*dnaQ*	1585662	1586633	-	DNA polymerase III subunit epsilon	2.05	9.45E-04	3.73E-02	-3035,-5289,-6320,-6641,-9129,-10817		+1409,+2225,+3402,+5547,+5672,+7655
Metobolism, transporter, and other											
jhp0099	*cysK*	110644	111561	-	cysteine synthase	2.46	5.38E-04	2.77E-02	-4253,-5073,-7195,-7494,-7934,-8655		+476,+524,+7472,+9656,+10355,+14846
jhp0180	*tpiA*	197063	197767	+	triosephosphate isomerase	2.47	3.57E-04	2.39E-02	-314,-1676,-5803,-6909,-9529,-12558		+5093,+7708,+8011,+8479,+10368,+11124
jhp0200		218958	220604	+	solute carrier family 13,sodium-dependent transporter	1.98	1.42E-03	4.75E-02	-542,-1145,-3057,-6644,-9385,-10067	219252,220110,220277	+264,+6024,+7786,+13763,+14906,+16129
jhp0342	*hofB*	373015	374454	+	Predicted outer membrane transport protein HofB	2.05	6.78E-04	3.24E-02	-1463,-3134,-4367,-5359,-7869,-9957		+588,+2912,+3117,+4547,+5789,+6052
jhp0384	*rpsO*	413760	414032	+	30S ribosomal protein S15	2.20	9.23E-04	3.73E-02	-432,-468,-8669,-14040,-14292,-16763		+742,+1883,+7776,+12049,+12264,+14570
jhp0423	*kefB*	455743	456993	-	glutathione-regulated potassium-efflux system protein	2.19	1.51E-03	4.83E-02	-65,-566,-1896,-2307,-3479,-4655	456167	+1201,+4679,+9634,+12305,+14494,+16389
jhp0545	*bioF*	593501	594622	-	8-amino-7-oxononanoate synthase	4.19	7.70E-11	1.03E-07	-5066,-5270,-5417,-6375,-6673,-9424	594156	+589,+3799,+7199,+8273,+8461,+10730
jhp0724	*lolC*	804721	805953	+	Predicted lipoprotein release system transmembrane protein	2.48	6.71E-05	7.64E-03	-2504,-2630,-4877,-5359,-13684,-13951	805233,805707,805854	+2196,+2641,+13328,+13798,+15155,+15573
jhp0769	*gatA*	846840	848201	-	aspartyl/glutamyl-tRNA amidotransferase subunit A	2.13	9.21E-04	3.73E-02	-1480,-2409,-3571,-3983,-5046,-5386	847084	+4851,+6863,+7028,+9870,+12971,+16468
jhp0781	*thiE*	859321	859974	-	thiamine-phosphate pyrophosphorylase	3.09	5.92E-05	7.64E-03	-716,-2176,-2590,-4635,-5504,-6595	859946,859793,859610	+4640,+5262,+5734,+6074,+7137,+7549
jhp0841	*pta*	920263	921822	-	phosphotransacetylase	2.00	1.15E-03	4.16E-02	-761,-2049,-3333,-4700,-5891,-7363	921536	+1609,+1835,+8654,+9097,+12275,+14594
jhp0980	*aroA*	1088513	1089802	+	3-phosphoshikimate 1-carboxyvinyltransferase	2.29	3.91E-04	2.39E-02	-4494,-5359,-6435,-8215,-8874,-8955	1089535	+112,+400,+1427,+2358,+2441,+3054
jhp1324	*kpsF*	1453242	1454231	+	Predicted polysialic acid capsule expression protein	3.61	3.78E-09	1.61E-06	-1625,-2791,-7288,-7657,-8766,-9353		+7193,+7902,+10051,+11292,+12286,+12705
Unknown function											
jhp0355		386564	386824	-	hypothetical protein	3.58	1.60E-08	3.58E-06	-967,-2530,-4358,-5278,-14608,-15431		+1103,+1210,+2246,+2420,+9227,+10399
jhp0440		475860	478598	+	hypothetical protein	2.50	1.67E-04	1.31E-02	-1305,-2752,-4237,-7338,-8832,-9195	477137	+944,+1541,+4108,+4988,+7793,+13259
jhp0555		607216	607785	+	hypothetical protein	2.45	4.01E-04	2.39E-02	-336,-920,-1950,-2186,-2589,-6885		+1705,+1906,+7473,+8500,+19576,+20337
jhp0619		687381	688148	-	hypothetical protein, predicted integral membrane protein	3.04	3.53E-04	2.39E-02	-1936,-3088,-7106,-8451,-9595,-10750		+3713,+4129,+6139,+6225,+8131,+8552

jhp0687		767426	768628	+	hypothetical protein	2.79	4.57E-06	7.66E-04	-767,-3485,-7607,-7961,-12266,-14704	768196	+112,+3641,+5859,+6186,+8033,+9336
jhp0986		1096410	1097078	+	hypothetical protein	4.12	2.02E-09	1.36E-06	-317,-368,-1409,-1658,-1973,-3335	1096445,1096493	+3565,+3581,+3634,+3895,+4519,+6294
jhp0987		1097080	1097838	+	hypothetical protein	4.00	4.79E-09	1.61E-06	-587,-635,-987,-1038,-2079,-2328		+2805,+2821,+2874,+3135,+3759,+5534
jhp1044		1157436	1160900	-	hypothetical protein	2.65	7.98E-05	7.64E-03	-762,-1554,-2466,-4624,-8164,-12590	1159381,1157580	+2079,+2700,+4012,+5812,+6840,+7792
jhp1319		1448519	1449139	+	hypothetical protein	3.67	7.46E-07	1.43E-04	-2565,-2934,-4043,-4630,-5517,-7955		+1312,+2478,+12285,+12994,+15143,+16384
**Repressed gene list**											
**ORF**	**Gene**	**Start**	**End**	**Strand**	**Description**	**log2 fold change (KO vs. WT)**	**p value**	**FDR**	**Distance to 5 nearest upstream TCANNNNNNTRG sites**	**TCANNNNNNTRG positions in coding region**	**Distance to 5 nearest downstream TCANNNNNNTRG sites**
DNA processing											
jhp0629	*hpy99IVM*	700587	701801	+	type II DNA modification enzyme	-3.76	6.92E-04	3.24E-02	-1603,-4178,-4573,-6132,-7322,-10700		+1724,+3952,+6651,+7152,+8997,+9063
jhp0931	*topA_3*	1032846	1034906	+	topoisomerase I	-4.37	4.27E-04	2.39E-02	-786,-2443,-2477,-2918,-4814,-5333	1033118,1034267	+273,+686,+4793,+5491,+7331,+7675
jhp1166	*ssb*	1299583	1300128	-	single-stranded DNA-binding protein	-3.45	7.24E-04	3.24E-02	-1496,-3340,-5076,-13422,-14199,-23258		+1244,+3776,+6511,+7429,+9805,+10132
Metobolism and other											
jhp0135	*fixP*	151681	152559	+	cytochrome oxidase (CBB3-type)	-5.94	5.30E-04	2.77E-02	-1215,-1452,-2531,-14240,-15086,-15664		+222,+2197,+2668,+3484,+5398,+5494
jhp0709	*amiA*	786502	787911	-	N-acetylmuramoyl-L-alanine amidase	-4.13	1.26E-03	4.44E-02	-2265,-3121,-3217,-3575,-5281,-5530		+2915,+3260,+4221,+5725,+7702,+7780
jhp0909	*gatC*	1005692	1005973	+	aspartyl/glutamyl-tRNA amidotransferase subunit C	-5.90	2.31E-05	3.44E-03	-1709,-3568,-5496,-6403,-8742,-9127		+1927,+2881,+4107,+7179,+9308,+10211
jhp0968	*cfa*	1072958	1074127	+	cyclopocyclopropane fatty acid synthase	-4.44	8.58E-05	7.68E-03	-831,-5025,-5375,-5970,-9402,-12694		+1243,+1651,+3224,+4108,+5280,+5431
jhp1039	*purB*	1151605	1152927	+	adenylosuccinate lyase	-5.59	1.26E-04	1.06E-02	-516,-1833,-9063,-10604,-10928,-11430		+2842,+3478,+4189,+5361,+10450,+12079
jhp1437	*hcpG*	1584853	1585623	+	Cysteine-rich protein G, related to amoxicillin resistance	-5.93	4.23E-04	2.39E-02	-1417,-2106,-2856,-4834,-5409,-5476		+141,+1185,+1657,+3199,+5793,+6378
Unknown function											
jhp0093		103627	104388	+	hypothetical protein	-4.19	1.47E-03	4.80E-02	-2421,-2985,-4215,-4909,-13452,-14022		+848,+1124,+1930,+3141,+6601,+8226
jhp0360		391140	391547	-	hypothetical protein	-4.33	1.03E-03	3.85E-02	-555,-9885,-10708,-12948,-15620,-17594	391182	+1786,+3349,+5679,+5786,+6822,+6996
jhp0816		894929	896773	-	hypothetical protein	-5.23	2.37E-04	1.77E-02	-2272,-2625,-3799,-3895,-6010,-6049	895374,895125	+259,+3553,+3778,+5413,+5600,+6046
jhp0928		1025324	1032019	+	hypothetical protein	-4.13	1.31E-03	4.52E-02	-2870,-8112,-8769,-9140,-10043,-12172	1025645,1026292,1027513,1028032,1029928,1030369,1030403	+41,+1099,+2248,+3160,+3573,+7680
jhp0934		1036277	1037296	-	hypothetical protein	-4.55	9.83E-04	3.77E-02	-1609,-2816,-4140,-5659,-7214,-8098		+2386,+6192,+7488,+7973,+8444,+8492
jhp1070		1183290	1185569	-	hypothetical protein	-5.12	7.92E-05	7.64E-03	-1161,-1950,-2188,-3085,-3729,-6379		+1337,+1595,+5254,+6642,+8389,+9800
jhp1113		1237715	1238866	-	hypothetical protein	-4.39	9.10E-04	3.73E-02	-775,-2692,-4531,-4882,-4979,-6467		+599,+2655,+3012,+3823,+4366,+4510
